# PRICKLE1 Interaction with SYNAPSIN I Reveals a Role in Autism Spectrum Disorders

**DOI:** 10.1371/journal.pone.0080737

**Published:** 2013-12-03

**Authors:** Lily Paemka, Vinit B. Mahajan, Jessica M. Skeie, Levi P. Sowers, Salleh N. Ehaideb, Pedro Gonzalez-Alegre, Toshikuni Sasaoka, Hirotaka Tao, Asuka Miyagi, Naoto Ueno, Keizo Takao, Tsuyoshi Miyakawa, Shu Wu, Benjamin W. Darbro, Polly J. Ferguson, Andrew A. Pieper, Jeremiah K. Britt, John A. Wemmie, Danielle S. Rudd, Thomas Wassink, Hatem El-Shanti, Heather C. Mefford, Gemma L. Carvill, J. Robert Manak, Alexander G. Bassuk

**Affiliations:** 1 The University of Iowa, Iowa City, Iowa, United States of America; 2 Department of Pediatrics, The University of Iowa, Iowa City, Iowa, United States of America; 3 Interdisciplinary Program in Genetics, The University of Iowa, Iowa City, Iowa, United States of America; 4 Department of Ophthalmology and Visual Sciences, The University of Iowa, Iowa City, Iowa, United States of America; 5 Department of Neurology, The University of Iowa, Iowa City, Iowa, United States of America; 6 Roy J. and Lucille A. Carver College of Medicine, The University of Iowa, Iowa City, Iowa, United States of America; 7 Department of Biology, The University of Iowa, Iowa City, Iowa, United States of America; 8 Interdisciplinary Graduate Program of Neuroscience, The University of Iowa, Iowa City, Iowa, United States of America; 9 Center for Bioresources, Brain Research Institute, Niigata University, Niigata, Japan; 10 Hospital for Sick Kids, University of Toronto, Toronto, Canada; 11 Developmental Biology Department, National Institute for Basic Biology, Okazaki City, Japan; 12 Section of Behavior Patterns, Center for Genetic Analysis of Behavior National Institute for Physiological Sciences, Okazaki, Japan; 13 Japan Science and Technology Agency, Kawaguchi-shi, Japan; 14 Division of Systems Medical Science, Institute for Comprehensive Medical Science, Fujita Health University, Toyoake, Japan; 15 Department of Pathology, The University of Iowa, Iowa City, Iowa, United States of America; 16 Interdisciplinary Graduate Program in Molecular and Cellular Biology, The University of Iowa, Iowa City, Iowa, United States of America; 17 Department of Psychiatry, The University of Iowa, Iowa City, Iowa, United States of America; 18 Shafallah Medical Genetics Center, Doha, Qatar; 19 Division of Genetic Medicine, Department of Pediatrics, University of Washington, Seattle, Washington, United States of America; The George Washington University, United States of America

## Abstract

The frequent comorbidity of Autism Spectrum Disorders (ASDs) with epilepsy suggests a shared underlying genetic susceptibility; several genes, when mutated, can contribute to both disorders. Recently, *PRICKLE1* missense mutations were found to segregate with ASD. However, the mechanism by which mutations in this gene might contribute to ASD is unknown. To elucidate the role of PRICKLE1 in ASDs, we carried out studies in *Prickle1^+/−^* mice and *Drosophila*, yeast, and neuronal cell lines. We show that mice with *Prickle1* mutations exhibit ASD-like behaviors. To find proteins that interact with PRICKLE1 in the central nervous system, we performed a yeast two-hybrid screen with a human brain cDNA library and isolated a peptide with homology to SYNAPSIN I (SYN1), a protein involved in synaptogenesis, synaptic vesicle formation, and regulation of neurotransmitter release. Endogenous Prickle1 and Syn1 co-localize in neurons and physically interact via the *SYN1* region mutated in ASD and epilepsy. Finally, a mutation in *PRICKLE1* disrupts its ability to increase the size of dense-core vesicles in PC12 cells. Taken together, these findings suggest *PRICKLE1* mutations contribute to ASD by disrupting the interaction with SYN1 and regulation of synaptic vesicles.

## Introduction

Autism Spectrum Disorder (ASD) is a term encompassing Asperger syndrome, classic autism, pervasive developmental disorder (PDD), PDD-NOS (not otherwise specified) [1]. ASD is evident by the age of three and characterized by a triad of symptoms: the absence of social interaction or responsiveness; absent or limited verbal communication; and restricted, stereotypical, and ritualized patterns of behavior and interests [2]. Since 1970, this genetic, neurodevelopmental disorder has become more prevalent in the USA, increasing from 1 in 2500, to 1 in 88 [3]. Up to 75% of patients suffer from mental retardation; 20–50% exhibit electroencephalographic abnormalities and up to 30% suffer from epilepsy [4]. Conversely, ASD is present in up to 30% of individuals with severe forms of epilepsy [5].

Epilepsy is a genetic disorder characterized by recurrent seizures and is associated with various genes, including several encoding ion channels [6], and others with no obvious ion channel function, such as *PRICKLE2* and *PRICKLE1* [7]. Recently, *PRICKLE1* missense mutations were found to segregate with ASD (Cukier, *et al.,* unpublished data presented at the American Society of Human Genetics 2012 Annual meeting). Although *PRICKLE1* mutations have been linked to epilepsy, the mechanism by which mutations in this gene might contribute to ASD is unknown. The main clues concerning the function of PRICKLE1 come from studies showing it is a member of the noncanonical WNT pathway, regulates planar cell polarity (PCP) and intracellular calcium release, and is characterized by PET and LIM domains [8], [9]. Further clues come from the obvious comorbidity of epilepsy with ASD, and the identification of ASD-predisposing mutations in genes encoding synaptic proteins (i.e., *SYN1*, *IL1RAPL1*, *RIMS3*, *NLGN3*, and *NLGN4*). These findings suggest *PRICKLE1* is among the predisposing genes which are shared by both disorders and destabilize synaptic homeostasis [10]–[13]. Taken together, these results have led to the hypothesis that synaptic dysregulation underlies both ASD and epilepsy [12].

In this study, we show that *Prickle1^+/−^* mice exhibit behaviors consistent with ASD. We also show that PRICKLE1 physically interacts with SYN1, a synaptic protein involved in synaptogenesis and synaptic vesicle trafficking. A mutation in PRICKLE1 disrupts its ability to increase dense-core vesicle size in stably transfected, inducible, PC12 cell lines. Taken together, these findings suggest that *PRICKLE1* is a novel, predisposing gene for ASD.

## Materials and Methods

### Mouse Behavioral Studies

Heterozygotes *Prickle1^+/−^* mice were produced as previously described [14]. Mice have been backcrossed onto C57/BL6>10 generations.

### Ethics Statement

All mouse work was done according to the requirements of the University of Iowa Office of the Institutional Animal Care and Use Committee. All protocols used were ethics board-approved. Personnel and investigators who handled the mice were properly trained and qualified. Animals were treated humanely, housed appropriately, and discomfort and distress were minimized during the experiments.

#### General health and neurological screening

Tests were carried out as previously described [15].

#### Hot plate test

A hot plate test was used to evaluate sensitivity to a painful stimulus. Mice (*Prickle^+/−^ n* = 16, controls *n* = 24) were placed on a hot plate (Columbus Instruments, Columbus, Ohio) at 55.0 (±0.3)°C, and latency to the first paw response was recorded. The paw response was a foot shake, a paw lick, or lifting both forepaws simultaneously.

#### Wire hang test

A wire hang test apparatus (Ohara & Co., Tokyo) was used to assess balance and grip strength in the test mice (*Prickle^+/−^ n* = 16, controls *n* = 24). The apparatus consists of a box (21.5×22×23 cm) with a wire mesh grid (10×10 cm) on its top, which can be inverted. The mouse was placed on the wire mesh, which was then inverted, causing the animal to grip the wire. Latency to fall was recorded, with a 60 sec cut-off time.

#### Grip strength test

A grip strength meter (Ohara & Co., Tokyo) was used to assess forelimb grip strength. Mice (*Prickle^+/−^ n* = 16, controls *n* = 24) were lifted and held by their tail so that their forepaws could grasp a wire grid. The mice were then gently pulled backward by the tail with their posture parallel to the surface of the table until they released the grid. The peak force applied by the forelimbs of the mouse was recorded in Newtons (N). Each mouse was tested three times and the highest value obtained was used for statistical analysis.

#### The home cage monitoring system

(11.8 cm×20.8 cm×14.5 cm) which provides food, water and bedding with an activity sensor (O’HARA & CO., LTD., Japan) was used to assess circadian rhythmic motor activity in wild-type and *Prickle1^+/−^* mouse littermates (6 each). The activity sensor linked to a computer program detects and measures motor activity by the number of signal changes in sensor elements from a pyroelectric infrared sensor, which detects the body heat when a test mouse moves. The program recorded cumulative number of counts every 10 minutes. All motions including horizontal locomotion, rearing, climbing on the lid, grooming, and other fine movements were detected. Animal behavior in the cage was recorded continuously using a CCD camera and infrared illuminations for 24 hours.

### Activity Test in Home Cage

Monitoring was performed in the home cage and conducted as previously described [16]. The system contains a home cage (29×18×12 cm) and a filtered cage top, separated by a 13-cm-high metal stand containing an infrared video camera fitted on top of the stand. Mice were placed in a home cage and outputs from the video cameras were fed into a Macintosh computer. Images from each cage were captured at a rate of one frame per second. We measured locomotor activity by quantifying the number of pixels that changed between each pair of successive frames. Analysis was performed automatically using Image SI software. The software was based on the public domain ImageJ program (http://rsb.info.nih.gov/ij/) and modified by the authors (available through O’Hara & Co., Tokyo, Japan).

### Social Interaction Paradigm

Open field chambers (40.6 cm×40.6 cm×36.8 cm) (San Diego Instruments, San Diego, CA) served as boxes for this assay. Sixteen wild-type C57/BL6 and eleven *Prickle1^+/−^* adult male (8–12 weeks old) mouse littermates from 3 different litters were tested. An unfamiliar, age-matched, wild-type conspecific mouse was placed in the test chamber and allowed to habituate for 10 minutes. A test mouse (either a wild-type or a *Prickle1^+/−^* mouse) was then introduced while being videotaped for 10 minutes. A blinded observer quantified sociability by scoring the amount of time the test mouse spent inspecting the novel mouse by sniffing, close huddling, and crawling over the other mouse.

### Context Fear Conditioning

Assays were carried out as previously described [17]. Mice were placed in a near-infrared video-equipped fear conditioning chamber (Med Associates, Inc., St. Albums, VT). Context fear conditioning training totaled 8 minutes. Mice explored the chamber for 3 minutes, and then 5 shocks (1 s, 0.75 mA) were administered through the grid flooring with an inter-trial interval (ITI) of 1 minute. Context-evoked freezing was tested by placing the mice back into the conditioning chamber for 6 min (minus foot shocks). Freezing was defined as an absence of movement other than respiration, and scored with VideoFreeze software (Med Associates, Inc.).

### TMT Evoked Freezing

TMT-evoked freezing was measured as described previously [18]. Briefly, mice were placed in a chamber with a beaker containing TMT (30 µl) (PheroTech). These behavioral chambers were distinct from the fear conditioning apparatus to avoid contaminating the fear conditioning equipment with TMT. Freezing was defined the same as for fear conditioning above and was scored from videotapes by an experimenter blinded to genotype.

### Auditory Cue Conditioning

Training (context A) totaled 14 minutes; mice explored the chamber for 3 minutes, and then 5 tones (80 dB, 3 kHz, 20 s) terminating with a shock (1 s, 0.75 mA) were presented with an ITI of 100 s. To assess conditioned freezing to the tone, mice were placed in a different context (a smooth floor and a black triangle insert were placed into the conditioning chamber with peppermint extract added to change odor), and freezing was assessed over 6 minutes, with the tone presentation occurring during minutes 4–6. To assess context-evoked freezing, mice were placed back into training environment (context A) without tone and without shock. Freezing was scored as in context fear conditioning over 5 minutes.

### Yeast Two-hybrid Analysis

Human PRICKLE1 (aa 1 to 827) was cloned into pB27 as a C-terminal fusion to LexA (N-LexA-PRICKLE1-C) and was used as a bait to screen a random-primed human adult and fetal brain cDNA library constructed into pP6. With the adult brain library, 53 million clones (5-fold the complexity of the library) were screened using a mating approach with HGX13 (Y187 ade2-101::loxP-kanMX-loxP, matα) and L40ΔGal4 (matα) yeast strains, as previously described [19]. 338 His+ colonies were selected on a medium lacking tryptophan, leucine, and histidine, and supplemented with 50 mM 3-aminotriazole to handle bait autoactivation. With the fetal brain library, 99 million clones (10-fold the complexity of the library) were screened. Here, 169 His+ colonies were selected. The prey fragments of positive clones were amplified by PCR and sequenced. The resulting sequences were used to identify the corresponding interacting proteins in the GenBank database (NCBI) using a fully automated procedure. A confidence score (PBS- Predicted Biological Score) was attributed to each interaction as previously described [20]. The final 9 protein sequences were aligned with Kalign to identify the consensus sequence.

The PBS relies on a local score, which takes into account the redundancy and independency of prey fragments, as well as the distribution of reading frames and stop codons in overlapping fragments, and a global score, which takes into account the interactions found in all the screens performed at Hybrigenics using the same library. The scores were divided into four categories, from A (highest confidence) to D (lowest confidence). E specifically flags interactions previously found several times in screens performed from the same organism; false-positives of the technique and were tagged as F. The PBS scores positively correlate with the biological significance of interactions [21], [22].

### Antibodies and Antibody-conjugated Agarose Beads

KLH-conjugated USIPP was used to raise anti-USIPP polyclonal antibodies in rabbits. Polyclonal anti-Prickle1 antibodies were produced as described [7]. The other antibodies were mouse anti-PSD-95 (Neuromab); polyclonal rabbit-anti Synapsin I (Abcam); mouse monoclonal anti-Actin (University of Iowa Developmental Studies Hybridoma Bank); mouse polyclonal anti-α-Tubulin (Sigma), mouse monoclonal anti-Myc (Sigma). Anti-rabbit, and anti-mouse horseradish peroxidase (HRP)-conjugated secondary antibodies (Thermo Scientific); agarose antibody-conjugated beads were anti-GFP (MBL Bion) and protein A/G beads (Pierce). Fluorophore-conjugated secondary antibodies used were AlexaFluor568-conjugated goat anti-rabbit IgG, AlexaFluor488-conjugated goat anti-mouse IgG, and AlexaFluor647-conjugated goat anti-mouse IgG (Invitrogen).

### Plasmids

USIPP cDNA was tagged with GFP at the C-terminus and cloned into the EcoRI (5′) and KpnI (3′) restriction sites of pCDNA3.1 vector. EGFP-ΔhSYN1; Human SYN1/USIPP homology region (aa 431–483) was tagged with eGFP at the N-terminus and cloned into the EcoRI (5′) and KpnI (3′) restriction sites of pcDNA3.1 vector.

Myc-REST and Flag-PRICKLE1 plasmids were previously described [7]. The N-terminus of PRICKLE1 (aa 1 to 313) and the C-terminus of PRICKLE1 (aa 314 to 831) were each Flag-tagged on the C-terminus and cloned into EcoRI (5′) and KpnI (3′) restriction sites of pcDNA3.1 vector. The other plasmids were Myc-DDK-SYNAPSIN Ia (OriGene) and eGFP-N1 (Clontech).

### Western Blots

Wild-type mouse brain, liver or kidney was lysed in ice-cold tissue lysis buffer (25 mM NaF, 10 mM Tris pH 7.4, 10 mM EGTA, 10 mM EDTA, 1X protease inhibitor cocktail and 10% sucrose) and centrifuged at 40,000 rpm for 30 minutes to separate supernatants. Equal amounts of proteins were resolved by sodium dodecyl sulfate-acrylamide gel electrophoresis (SDS-PAGE) in 4–20% acrylamide gels (pre-cast gels, Biorad) and transferred onto PVDF membrane for 3 hours. The membrane was blocked in 5% non-fat milk for 2 hours at room temperature followed by incubation in anti-USIPP antibody (1∶500) overnight at 4°C. The membrane was then washed in TBST (Tris-buffered saline with Tween-20) at room temperature, followed by incubation in HRP-conjugated goat-anti-rabbit antibody (1∶10 000) at room temperature for 2 hours. The blots were developed using ECL detection kit (Thermo Scientific) after washing as per the manufacturer’s instructions, and the signals were captured on X-ray films.

### Coimmunoprecipitation

Coimmunoprecipitations were carried out as previously described [7] in HEK293 cells [23]. HEK293 cells were co-transfected with GFP-USIPP and Flag-Prickle1, Flag-NPrickle1 or Flag-CPrickle1 with Polyfect (Qiagen), according to the manufacturer’s protocol. Cells were lysed after 48-hrs in ice-cold NET-100 buffer (Tris 50 mM, NaCl 100 mM, EDTA 5 mM supplemented with protease inhibitor (1X EDTA–free complete mini tabs protease inhibitor cocktail (Roche)). Lysates were immunoprecipitated overnight with anti-GFP beads at 4°C. Beads were washed for 5 minutes×5 times in ice-cold NET-100 buffer + protease inhibitor. Bound complexes were eluted with 2X Laemmli buffer at 100°C for 5 minutes, resolved by SDS-PAGE in 4–20% acrylamide gel, transferred onto PVDF membrane and then subjected to anti-Flag Western Blot analysis (1∶1000). HRP-conjugated goat anti-mouse secondary antibody was used at 1∶10 000 dilution.

#### Endogenous Prickle1 and Synapsin I coimmunoprecipitation

Wild-type brain lysate was incubated with protein A/G agarose beads and USIPP immune serum overnight at 4°C. Beads were washed 5×5 minutes in ice-cold NET-100 buffer supplemented with protease inhibitor. Bound complexes were eluted with 2X Laemmli buffer, resolved by SDS-PAGE and subjected to anti-Prickle1 Western Blot analyses (1∶200). HRP-conjugated goat anti-rabbit secondary antibody was used at 1: 10 000 dilution. The membrane was stripped afterwards and re-probed with anti-Synapsin I antibodies to verify immunoprecipitation of Synapsin I. Here also, HRP-conjugated goat anti-rabbit secondary antibody was used at 1: 10 000 dilution against anti-Synapsin I. In reverse, wild-type brain lysate was immunoprecipitated with protein A/G agarose beads and anti-Prickle1 antibodies overnight at 4°C. Immunoprecipitates were resolved by SDS-PAGE and subjected to anti-Synapsin I Western blots (1∶1000).

### Mass Spectrometry LC-MS/MS

#### SDS-PAGE

20 µL of the clarified, soluble protein solution was added to denaturing SDS-PAGE loading buffer (containing glycerin, beta-mercaptoethanol, and SDS in Tris buffer) and boiled for five minutes in preparation for electrophoresis. Bio-Rad precast 4–20% Tris-HCl gradient SDS-PAGE gels were run at 150 V for 45 minutes. Gels were then stained with Bio-Rad Flamingo fluorescent stain and imaged using a UVP PhotoDoc-It UV Imaging System (Upland, CA).

#### LC-MS/MS

Bands from the gel were cut out and placed into a prewashed Eppendorf tube. A blank section of gel was cut out as a negative background control. 250 µl 50% H_2_O/50% acetonitrile was added for 5 minutes and then removed. 250 µl 50% CH_3_CN/100 mM NH_4_HCO_3_ (0.158 g/20 ml) was added to all samples. Gel bands were cut into smaller pieces using a pair of tweezers and washed for 30 minutes at room temperature and then the wash was removed. CH_3_CN was added and incubated with the gel pieces for 30 minutes at room temperature and then discarded. Next, 250 µl 100 mM NH_4_HCO_3_ was added to the gel pieces. Gel pieces were completely dried using a speedvac and then 10 mM DTT solution was added to cover the gel pieces and heated for 1 hour at 56°C. Samples were cooled to room temperature and the DTT solution was replaced with an equal volume of 55 mM iodoacetamide solution. Proteins were incubated for 45 minutes at room temperature in the dark with occasional vortexing. Gel pieces were rehydrated with 100 µl of 25 mM ammonium bicarbonate, pH 8, for 10 min while vortexing, and dehydrated with 100 µl of 25 mM ammonium bicarbonate/50% acetonitrile. This step was performed two times. The liquid phase was removed and gel pieces dried to completion in a vacuum centrifuge. An appropriate amount of ice-cold trypsin solution in 25 mM NH_4_HCO_3_ was added to all samples and the blank. For strong bands, 10 ng/µl trypsin was added. For light bands, 8 ng/µl trypsin was added. Tubes were on ice for 20 minutes as the enzyme/buffer solution to absorb to swell the gel pieces. An additional 20 µl of chilled 25 mM NH_4_HCO_3_ that does not contain enzyme was added to the gel pieces and incubated at 37°C for 16–24 hours.

0.5% FA, 5% CH_3_CN was added to quench the digest and then the gel pieces were extracted by centrifugation at 1100×g for 6.5 minutes. The supernatant was removed containing the peptides and stored in Eppendorf tubes. A second extraction was performed by adding 50 µl 0.1% FA, 50% CH_3_CN. This step was repeated and the supernatants from all three extractions were combined. Supernatants were concentrated down to 4 µl left in tube using a Speedvac. Finally, 8 µl of 5%CH3CN/0.1% formic acid was added to each sample.

Using an Dionex 3000 nanoRSLC series HPLC system (Thermo-Electron, USA), 4 uL of recovered peptides were loaded at 2 uL/min onto a 200 um id by 2.5 cm precolumn (New Objective) packed with 5 um YMC ODS-A C18 beads (Waters, Milford, MA, USA).

Following an on-line desalting step, trap flow was rerouted through a self-packed 75 um id×9 cm analytical column containing 3 um Halo particles (Advanced Material Designs, with 300 Angstrom pore size) restricted by a distal spray opening 8 to 10 microns in diameter at approximately 200 nL/min using a 5–80% gradient of ACN (Honeywell) with 0.1% FA (Pierce) for 1 h. The LC effluent was electrosprayed into an LTQ linear ion-trap mass spectrometer (Thermo-Electron, USA). MS/MS spectra were acquired in a data-dependent acquisition mode that automatically selected and fragmented the six most intense peaks from each MS spectrum generated. Peptide and MS/MS mass tolerances were set to 1.8 and 0.4 Da, respectively.

#### Data analysis

MS/MS data were then analyzed and matched to mouse protein sequences in the Swiss Prot and TrEMBL database of Nov 9, 2012 using the MASCOT 2.4 database search engine (Matrix Science, UK) with carbamidomethyl as a fixed modification and oxidation as a single variable modification. Mass window for the parent ions were set to 1.8 m/z and 0.4 m/z for MS/MS data. The same spectra were searched using similar restrictions with the SpectrumMill algorithm (Agilent) and alignments were merged and curated using Scaffold v3.6.4. A minimum peptide ion score cut-off of 9 was set in SpectrumMill and presence of at least six consecutive y- or b-ions was required. Alignments reported from Scaffold were restricted to a false discover rate of less than 1%, with peptide and protein confidence >90% and at least two unique peptides required.

### Immunohistochemistry

#### Mouse brain sections

Procedures were performed as previously described [7], [24]. Harvested wild-type brain was fixed in 4% paraformaldehyde (PFA) and sectioned with a vibratome, permeabilized with 0.5% Triton X-100 PBS and then washed in Ca^2+^ and Mg^2+^-free phosphate-buffered saline (PBS). The sections were incubated in blocking solution consisting of 2% bovine serum albumen (BSA), 0.5% Triton X-100, and 1X PBS at a pH of 7. Floating 100 µm-thick sections were immunostained with both mouse anti-PSD95 (1∶50) and rabbit anti-USIPP (1∶50) for 4 hours, followed by goat anti-rabbit AlexaFluor488 and goat anti-mouse AlexaFluor568 (1∶200). Confocal images were captured with a Zeiss 710 microscope at 63X magnification.

For the co-labeling of anti-USIPP and anti-Synapsin I, blocked hippocampal sections were incubated in anti-Synapsin I (1∶100) overnight followed by goat anti-rabbit AlexaFluor488 (1∶200). The sections were then re-blocked for 2 hours at room temperature and then incubated in anti-USIPP (1∶100) antibody followed by goat anti-rabbit AlexaFluor568 (1∶200). For single staining, samples were incubated in either anti-Synapsin I or anti-USIPP antibodies overnight followed by incubation in goat anti-rabbit Fluor488 or goat anti-rabbit Fluor568 respectively. Confocal images were captured with a Zeiss 710 microscope at 63X magnification.

#### Mouse hippocampal neurons

Mouse hippocampal neurons were cultured as described from P0–P2 wild-type pups [25]. The dissected hippocampus was dissociated and then plated onto 35 mm poly-L-ornithine and laminin-coated glass coverslips. The cells were incubated in Neurobasal A supplemented with B27, 0.5 mM glutamine, 5% horse serum, and 10 mM HEPES for 4 hours. The medium then was then replaced with serum-free medium and maintained in a 5% CO_2_ incubator at 37°C. One-third of the medium was changed once a week. Cultured neurons were fixed on day 10 of culturing and then incubated in anti-Prickle1 (1∶400) and anti-Synapsin I (1∶500) antibodies overnight at 4°C, followed by AlexaFluor488 goat anti-rabbit (1∶1000) and AlexaFluor568 goat anti-mouse (1∶1000). Confocal images were obtained with a Nikon AIR.

### Construction of the UAS-EGFP-Pk

The 2.9 kb pk cDNA construct was commercially synthesized (GeneArt, Invitrogen) using the published cDNA sequence (NM_165508.2 GI:442622668). The 5′ UTR was omitted to place the initiation codon in frame for Gateway recombination, and the *pk* stop codon was included for proper protein termination. The construct was originally prepared as a cloned product in the pMK vector. EcoRI restriction enzyme sites were included outside the coding region at both ends of the construct to allow direct, in-frame subcloning into the pCR8-GW entry vector (Invitrogen). Integrity of the *pk* entry clone was confirmed via limited sequencing and restriction digestion. An LR clonase reaction (Invitrogen) was then performed to recombine the N-terminal *pk* entry vector with a UASt-EGFP (pTGW) destination vector (Terrence Murphy, Carnegie Institution). An open reading frame from the fluorescent tag into the *pk* coding region was confirmed by Sanger sequencing. The pUAST-EGFP-pk construct was submitted to Model System Genomics (Duke University) for *w^1118^* embryo injection. Individual transformants (*w+*) were balanced in *w-* background balancer stocks (Murphy, T.D., *et al*. Construction and application of a set of Gateway^™^ vectors for expression of tagged proteins in *Drosophila*, unpublished data).

#### Drosophila neuron immunohistochemistry

UAS-EGFP-pk flies were crossed to flies harboring the C155 GAL4 driver (an *elav* transgene located on the X chromosome used to drive the EGFP-pk in all neurons). F1 wandering third-instar larvae were dissected and fixed with 4% paraformaldehyde, washed with PBST (phosphate-buffered saline (PBS) with 0.1% Tween 20) and blocked with PBSTB (PBST with 0.1% BSA). Larvae were stained with rabbit anti-GFP (1∶500; Invitrogen, catalog number A-6455) and mouse anti-Synapsin 11C3 (1∶50; Developmental Studies Hybridoma Bank, University of Iowa).

### Drosophila Lines and Genetics

The Oregon-R control flies were reported previously [24]. The *Synapsin* homozygous mutant flies (w^*^ P{GawB}MzVum; *Syn^97^*) were obtained from the Bloomington *Drosophila* Stock Center (stock number 29031). The *Syn^97^* allele is a loss-of-function amorphic allele caused by a P-element-mediated deletion of the 5′ end of the gene. All fly lines were subjected to identical culture conditions and standard *Drosophila* cornmeal food media.

#### Modified bang-sensitivity behavioral assay

For climbing analysis, ten flies (five males and five females) were collected immediately after eclosion under CO_2_ and placed in a food vial and kept in a 25°C incubator. Flies were transferred to a new food vial every two days and were aged for a total of 4 days. At day 4, flies were transferred to clear glass vials and subjected to a mechanical vortexing force (control and mutant flies were age matched and vortexed at the same time) for 20 seconds using matched Fisher Scientific Vortex Mixers set to the highest setting (power = 10). After vortexing, the flies were digitally recorded using a Sony camcorder and their recovery was assessed in 5 second intervals up to 25 seconds. Only flies that left the bottom of the vial at each interval were considered as recovered flies, while flies that stayed at the bottom of the vial were considered non-recovered flies. All scoring was statistically assisted using Fisher’s exact test.

### Generation of Inducible PC12 Cells

We used PC12 cells–a model system for investigating neuronal differentiation in cultured cells [26]. A PC6-3 (sub-clone of PC12 cells) clonal line stably expressing the tet-repressor pcDNA6/TR (PC6-3/TR) was generously provided by Pedro Gonzalez-Alegre (University of Iowa, Iowa City). Clonal cell lines that inducibly express GFP, GFP-PRICKLE1 or GFP-PRICKLE1R104Q were generated as previously described [27]. In brief, GFP, GFP-PRICKLE1, and GFP-PRICKLE1R104Q cDNAs were cloned into EcoRI and XhoI restriction sites of pcDNA_5_TO (Invitrogen). The plasmids were transfected with Lipofectamine 2000 (Qiagen) into the PC6-3/TR cells according to the manufacturer’s instructions. Transfected cells were selected in medium containing hygromycin (100 µg/ml) and blasticidin (5 µg/ml). Twenty-four clones were selected for each plasmid and screened for inducibility, by adding 1.5 µg/ml doxycycline to the media and measuring transgene expression by fluorescence microscopy and Western blotting using an anti-GFP antibody (1∶1000).

### Transmission Electron Microscopy (TEM)

The stable cell lines were processed as previously described [27], [28], differentiated in low serum medium (with 100 ng/ml NGF) with doxycycline and then fixed in 2.5% glutaraldehyde after 7 days. Cells were processed in Eponate 812, cut into 80 nm sections and then stained with 2.5% uranyl acetate and lead acetate. Images were captured on a JEOL JEM-1230 Transmission Electron Microscope with a Gatan UltraScan 1000 2 k×2 k CCD camera, at 5000X magnification.

### Dense-Core Vesicle (DCV) Analysis

DCV sizes were measured as previously described [29]. Here, random (scorer blinded) sections of the cytoplasm were photographed and DCVs located away from the Golgi apparatus were measured [29]. Using ImageJ, the surface areas of DCVs were measured. For eGFP, NGF + Dox n = 36; for GFP-PK1, NGF + DOX n = 61; and GFP-PK1R104Q NGF + DOX n = 86. Using Microsoft Excel, the vesicle size for each condition was averaged and represented by bar graphs.

### Immunocytochemistry

PC12 cells stably expressing GFP, GFP-Prickle1 or GFP-Prickle1R104Q were treated with NGF and doxycycline for 48 hours. Cells were fixed in 4% formaldehyde for 15 minutes, washed in PBS and then permeabilized in 0.2% Triton X-100/PBS for 20 minutes. After washing and blocking in 5% BSA for 1 hour, cells were incubated in mouse α-Tubulin (1∶5000) and rabbit anti-Synapsin I (1∶70) overnight. After washing, cells were incubated in goat anti-rabbit AlexaFluor586 and goat anti-mouse AlexaFluor647 against Synapsin I and α-Tubulin respectively. Confocal images were taken on a Zeiss 710 at 63X magnification.

### Protein Accession Numbers

Human-Synapsin IA NP_008881.2 GI: 19924099; mouse-Synapsin I NP_038708.3 GI: 160707901; human-Prickle1 NP_001138354.1 GI: 23308518; *Drosophila* prickle NM_165508.2 GI:442622668).

### PSI Blast

USIPP sequence was entered in PSI and blasted against all human protein sequences. One thousand hits were generated and narrowed down to 230 brain-expressed proteins.

### Statistical Analyses

Using SPSS, ANOVA with Bonferroni correction was used to compare the sizes of dense core vesicles in PC12 cells. For memory, fear, and TMT tests, Students’ *t*-test was used for evaluating statistical significance. Grip strength, wire-hang, and general health and neurological screening data were analyzed by one-way analysis of variance (ANOVA). Activity data was analyzed by two-way repeated measures ANOVA. Fisher’s exact test was used to analyze *Drosophila* Bang-sensitivity assay scoring data. The criterion for statistical analyses was set at <0.05.

## Results

### *Prickle1^+/−^* Mice Exhibited ASD-like Behaviors

The etiology of ASD remains largely unknown, since most cases are likely caused by a combination of environmental and genetic components [30]. Although it may be difficult to draw causal links to behavior, insights may be gained by manipulating highly penetrant, single genes in mouse models [31]. We therefore carried out behavioral studies in *Prickle1^+/−^* male mice (homozygous mutant mice die *in utero*), and compared them to wild-type controls to assess whether loss of one copy of *PRICKLE1* leads to an ASD-like phenotype.

#### Altered social behavior

**Figure 1:**
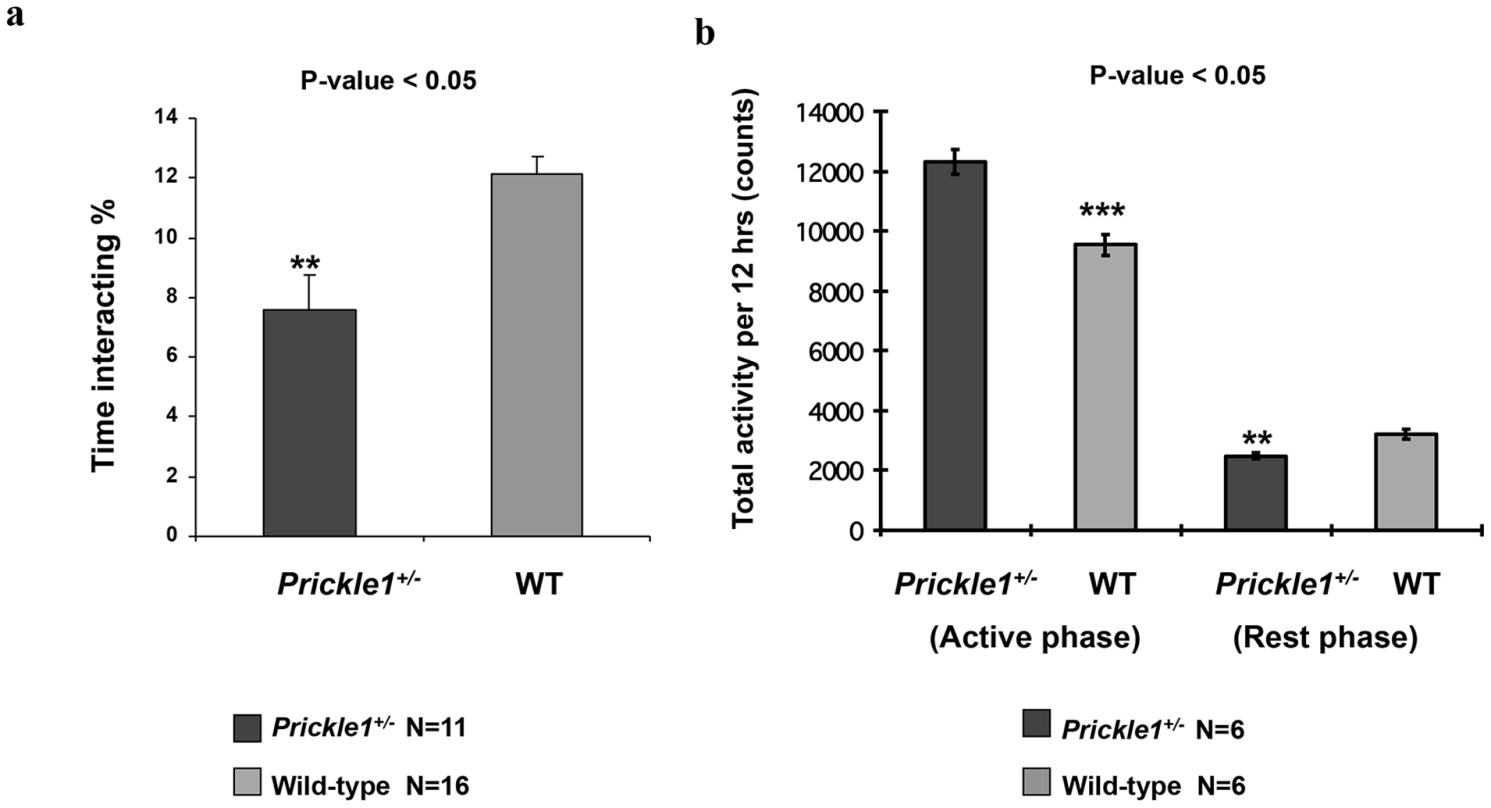
*Prickle1^+/−^* mice exhibited abnormal activity consistent with ASD-like behavior. (**a**) Open field chambers were used to carry out freely moving social assays to measure sociability in the *Prickle1^+/−^* mice. Eleven *Prickle1^+/−^* and sixteen wild-type C57/BL6 adult mice at 8–12 weeks old were tested. An unfamiliar, age-matched, wild-type mouse habituated the test chamber for 10 minutes. Each test mouse was then introduced and videotaped for 10 minutes. Sociability was scored by the amount of time the test mouse spent inspecting the novel mouse by sniffing, close huddling, and crawling over the other mouse. Compared to the WT, heterozygous mice exhibited deficiencies in social behavior (**p-value = 0.00084). (**b**) *Prickle1^+/−^* mice exhibited altered circadian rhythms. The home cage monitoring system, equipped with an activity sensor was used in this assay. Six each of WT and heterozygote *Prickle1^+/−^* littermates were tested. The activity sensor linked to a computer program detected and measured motor activity when the test mouse moved. Motions such as horizontal locomotion, rearing, climbing on the lid, grooming, and other fine movements were recorded continuously using a CCD camera and infrared illuminations for 24 hours. During the active phase, *Prickle1^+/−^* mice were significantly more active than the WT (***p-value = 0.0000002417); however, in the rest phase *Prickle1^+/−^* mice were significantly less active than their WT counterparts (**p-value = 0.00036).

A freely moving social-interaction assay measured sociability, a characteristic used to assess autistic-like behavior in mice [32], [33]. As seen in Figure 1a, *Prickle1^+/−^* mice spent significantly less time socializing with an unfamiliar mouse (indicated by sniffing; p-value = 0.00084). In contrast, the mutant mice displayed normal total locomotion, eating, drinking, cued and context fear conditioning, and a normal reaction to the odor of 2,3,5-Trimethyl-3-thiazoline (TMT). Home cage behaviors and general health conditions of both WT controls and *Prickle1^+/−^* mice appeared normal. Also, body weight and temperature were not significantly different between the genotypes (Supporting figures 1–3).

**Figure 2:**
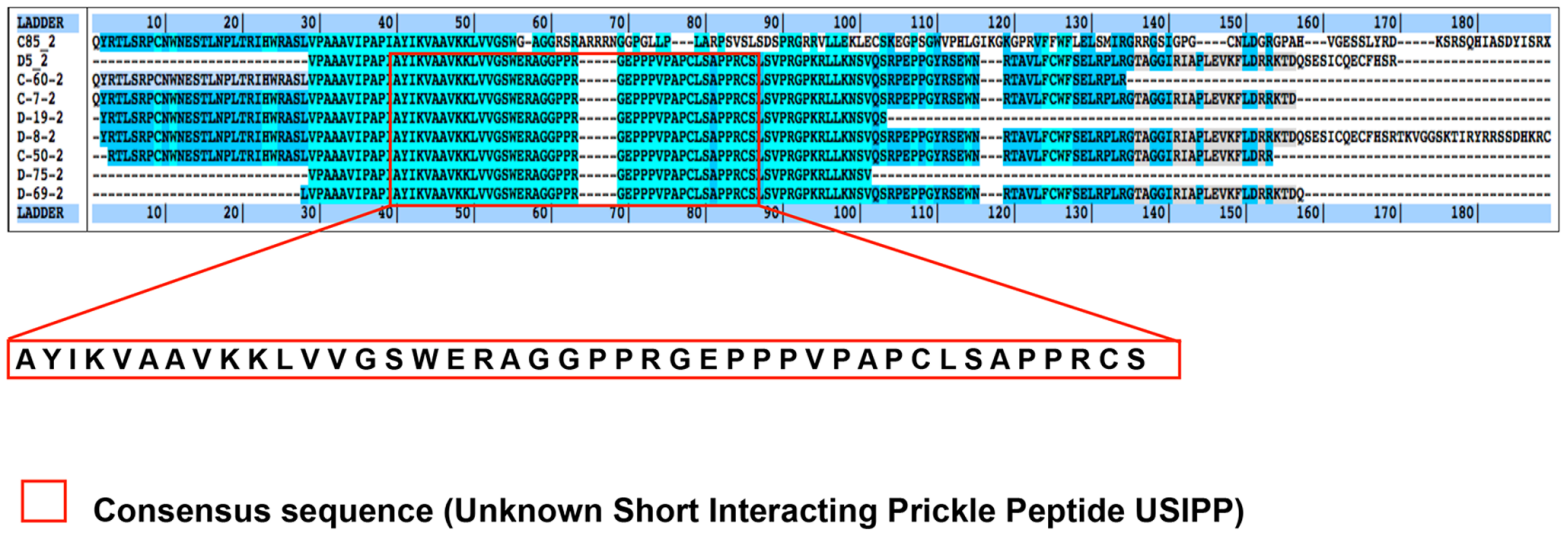
PRICKLE1 Yeast two-hybrid screen isolates Unknown Short Interacting Prickle1 Peptide (USIPP). Using Human PRICKLE1 (aa 1 to 827) N-LexA-PRICKLE1-C cloned into pB27 vector as bait, a yeast two-hybrid assay was used to screen random-primed adult and fetal cDNA libraries constructed into the pP6 vector. 53 million clones (adult brain library) and 99 million clones (fetal brain library) were screened using a mating approach and nutritional selection in media lacking tryptophan, leucine, and histidine. Applying a high Predicted Biological Score (PBS), nine final sequences were identified out of a total of 507 positive clones selected. Multiple sequence alignment of the peptides with Kalign® highlights an unknown 42-residue consensus peptide sequence (USIPP).

**Figure 3:**
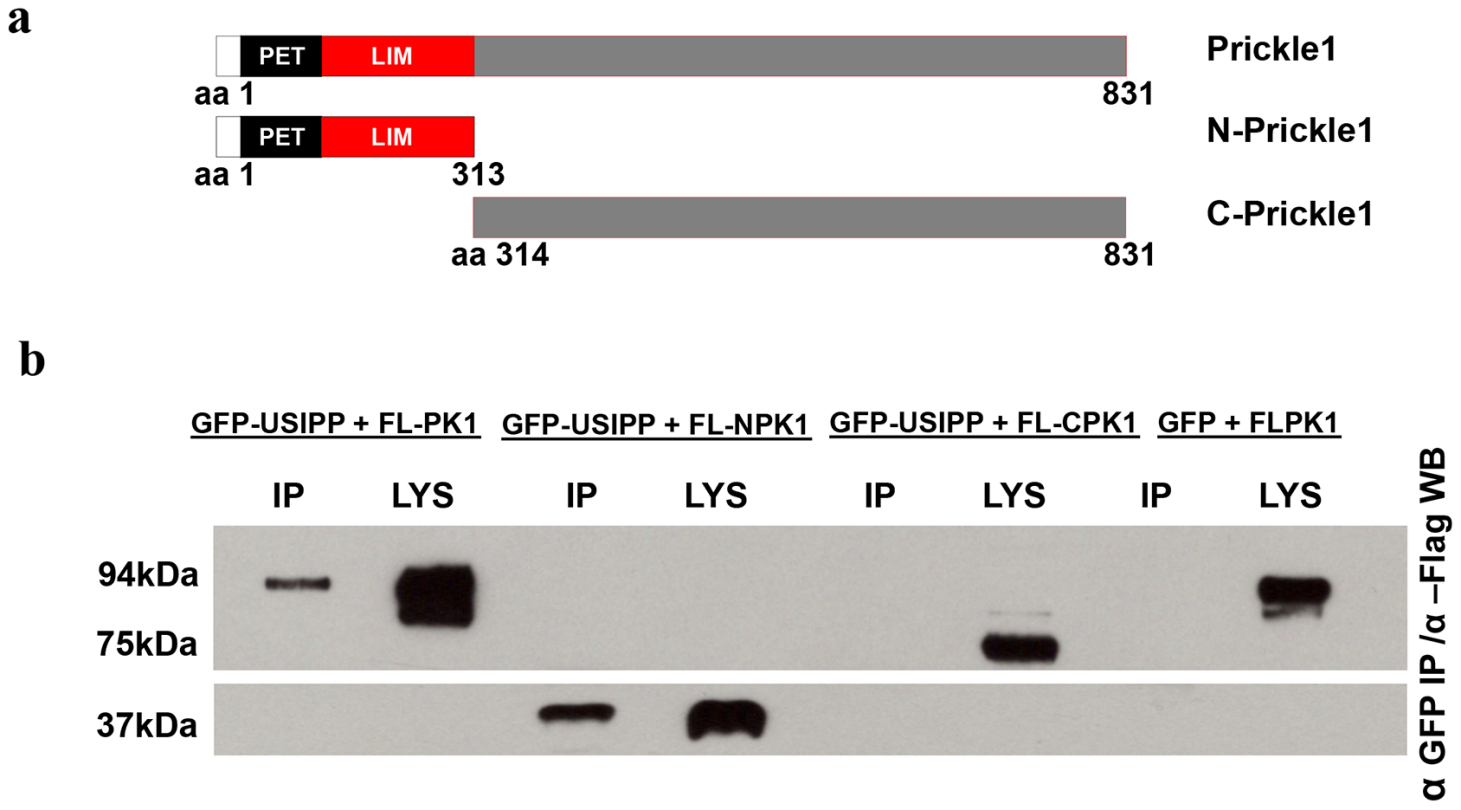
Coimmunoprecipitation of full-length and N-terminus PRICKLE1 with USIPP. (**a**) Flag-tagged Full-length PRICKLE1 (aa 1 to 831), PRICKLE1 N-terminus (aa 1 to 313) and PRICKLE1 C-terminus (aa 314 to 831). The N-terminus includes the PET/LIM domains: a region known to mediate PRICKLE1 protein-protein interactions. (**b**) HEK293 cells were co-transfected with the indicated constructs (Flag-PRICKLE1+ GFP-USIPP, Flag-NPRICKLE1+ GFP-USIPP, Flag-CPRICKLE1+ GFP-USIPP or Flag-PRICKLE1+ GFP) and lysed after a 48-hour incubation period in NET-100 buffer. Lysates were immunoprecipitated with agarose-conjugated anti-GFP beads and eluted in Laemmli buffer. Immunoprecipitates were resolved by SDS-PAGE and subjected to anti-flag Western blot analysis.

#### PRICKLE1 mutant mice have disrupted circadian rhythms

Sleep disruption and a perturbed circadian rhythm are characteristic features associated with ASD-like behaviors in mice, rats, and humans [34], [35]. Using a home-monitoring system, the activities of test mice were recorded for 24 hours under light- and dark-cycle conditions (Fig. 1b). Compared to wild-type littermates, *Prickle1^+/−^* mice exhibited disrupted circadian rhythms, during both the dark and light phases (Fig. 1b). During the dark phase, which is the active phase for mice, *Prickle1^+/−^* mice were significantly more active (p = 2.417×10^−7^), while during the light phase (rest phase) the *Prickle1^+/−^* mice were significantly less active (p = 0.00036). Other unusual behaviors included repetitive behavior, as evidenced by repeated jumping in some *Prickle1^+/−^* mice (see Video S1). These findings suggest that loss of one *Prickle1* allele contributes to ASD-like behavior.

#### A PRICKLE1 yeast two-hybrid (Y2H) screen isolated the Unknown Short Interacting Prickle-1 Peptide (USIPP)

To investigate the mechanism by which PRICKLE1 contributes to ASD, human adult and fetal brain cDNA libraries were screened for PRICKLE1-interacting partners using a Y2H assay. Since the PRICKLE1 CIIS motif (amino acids 828 to 831) localizes the protein to the nuclear membrane [36], this motif was excluded from the screen to ensure cytoplasmic localization. Amino acids 1 through 827 (N-LexA-PRICKLE1-C) were used as bait. Several adult and fetal brain cDNAs from positive clones were identified as arising from the human 18S non-coding RNA (Tables 1A and 1B in File S1). Analysis of the various 18S RNA-encoding clones demonstrated that they were represented by nine slightly different clones (see Methods). These identified 18S non-coding RNA sequences have never been recovered in the over 2000 Y2H screens previously performed (Hybrigenics Services, Paris, France), suggesting that their selection was not simply an artifact of the screen, and that a translated product from these clones specifically associated with PRICKLE1. Alignment of the amino acid sequence encoded by these clones revealed a 42-residue consensus sequence, which we named Unknown Short Interacting Prickle Peptide (USIPP), since it had no known exact match in the human proteome (Fig. 2).

#### USIPP physically interacts with full-length and the N-terminus of PRICKLE1

In the Y2H, PRICKLE1 interacted with USIPP robustly; however, non-specific interactions can cause these screens to produce false positive results [37]. Therefore, the interaction was tested by co-immunoprecipitation assays in HEK293 cells. Again, USIPP physically interacted with PRICKLE1 (Fig. 3b). To map the region on PRICKLE1 that interacted with USIPP, recombinant vectors expressing GFP-USIPP were cotransfected with vectors expressing full-length PRICKLE1 (amino acids 1 to 831), the PRICKLE1 N-terminus (amino acids 1 to 313) or the PRICKLE1 C-terminus (amino acids 314 to 831; illustrated in Fig. 3a); and complexes were immunoprecipitated via the GFP tag. These experiments showed USIPP interacted specifically with the PRICKLE1 N-terminus (Fig. 3b). This region of PRICKLE1 includes the PET/LIM domains, a region previously shown to mediate Prickle1 protein-protein interactions [9], [38].

### PSI Blast

The human 18S RNA is not known to be translated; and the USIPP protein does not exactly match any sequence in the human proteome. Nevertheless, the Y2H findings and the specificity of the physical interactions strongly suggested the human proteome expresses a protein with structural homology to USIPP. Alignment of USIPP peptide against the top 1000 hits in the entire human proteome by PSI-blast (http://www.ebi.ac.uk/Tools/sss/psiblast/) peptide homology search yielded a list of 230 potential candidates which are proteins expressed in the brain (Table 2 in File S1).

#### USIPP antibodies recognize a brain-specific 74 kDa protein

**Figure 4:**
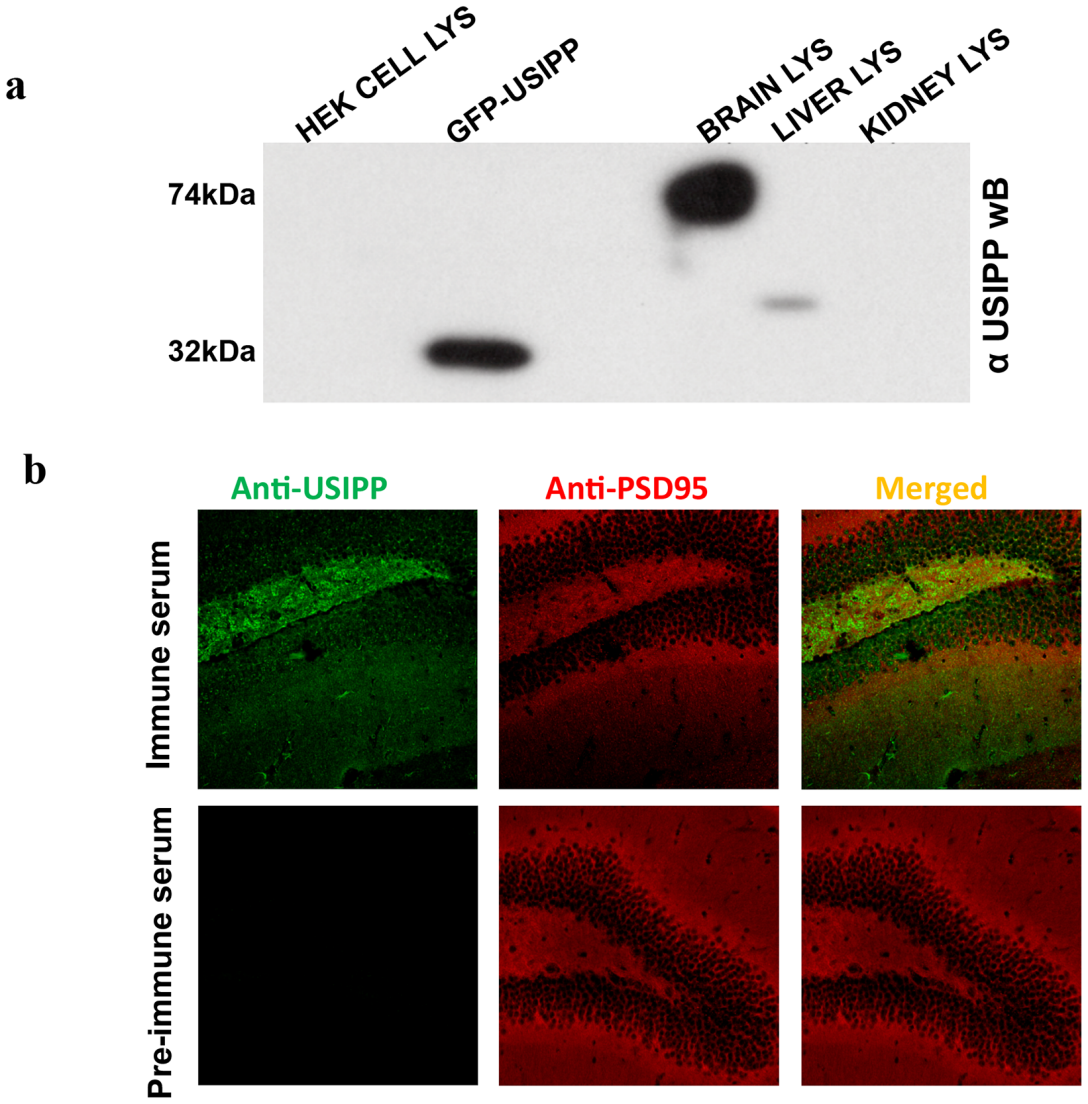
USIPP antibody recognizes a brain-specific, 74 kDa protein. (**a**) Anti-USIPP immunoblot shows an unknown brain-specific 74 kDa in a wild-type mouse. Lysates prepared from wild-type mouse brain, kidney or liver were resolved by SDS-PAGE and subjected to anti-USIPP Western blot analysis. (**b**) Confocal images show USIPP antibody immunostaining endogenous protein in dentate gyrus region of a wild-type mouse hippocampus. Sections of WT mouse hippocampi were incubated in anti-PSD-95 and rabbit pre-immune serum or immune USIPP serum followed by red AlexaFluor568 goat anti-mouse (PSD-95) and green AlexaFluor488 goat anti-rabbit (USIPP) secondary antibodies. Confocal images were captured with a Zeiss 710 microscope. The size markers correspond to 100 µm.

Operating under the premise that USIPP was structurally similar to a known brain protein, we raised rabbit polyclonal antibodies against USIPP. The antigen used to raise the antibodies was composed of amino acids 1 to 24 of USIPP because the remainder of the sequence contains multiple proline residues, which may cause low antigenicity. Western blots of cell lysates showed the anti-USIPP antibodies recognized a brain-specific, 74 kDa protein (Fig. 4a). Confocal images in Fig. 4b show immunostaining with the antibodies detected expression of an unknown, endogenous protein in the dentate gyrus of the mouse hippocampus, a region that also expresses PRICKLE1 and PRICKLE2 [7], [39].

#### SYN1 is immunoprecipitated by USIPP antibodies

**Figure 5:**
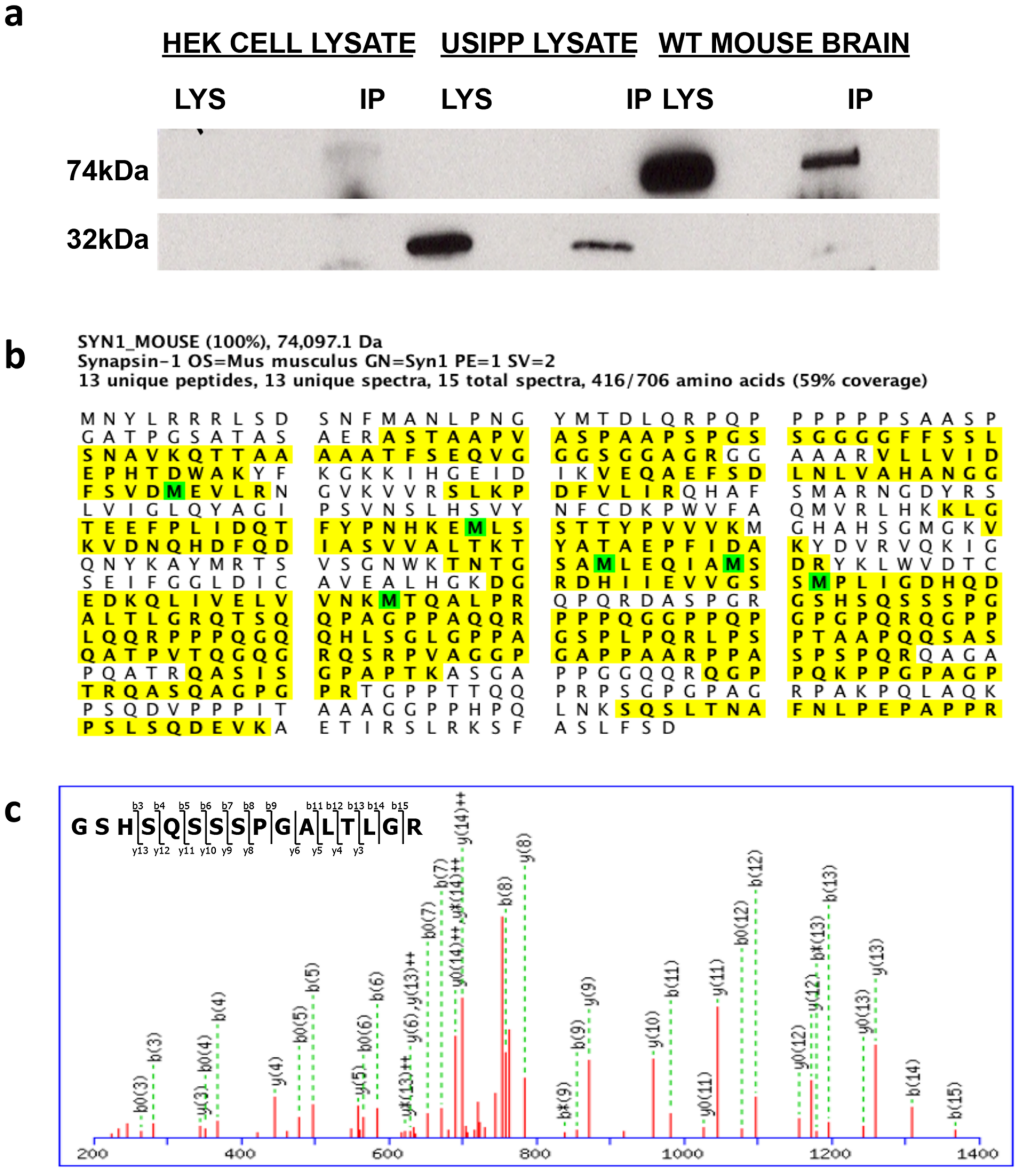
Protein immunoprecipitated with anti-USIPP antibodies identified as SYNAPSIN I. (**a**) Wild-type mouse brain was lysed in ice-cold tissue lysis buffer. Lysates were immunoprecipitated overnight with A/G beads and anti-USIPP antibodies. The immunoprecipitate was resolved and verified with anti-USIPP Western blotting. (**b**) Immunoprecipitated protein was analyzed using mass spectrometry. Thirteen unique peptides that matched SYNAPSIN I protein were identified. The 13 unique proteins identified by the mass spectrometer represented 59% coverage of SYN1. (**c**) Representative results of the amino acid AMUs and identified mass-to-ion ratio chromatogram of a single identified peptide of SYNAPSIN I region homologous to USIPP.

Proteins from mouse brain immunoprecipitated with the anti-USIPP were separated by SDS-PAGE and verified by Western blotting (Fig. 5a). Bands from brain extracts were excised and analyzed using mass spectrometry, which recovered thirteen unique peptide matches to the SYN1 protein sequence. SYN1 peptides were absent from the control lane. These unique peptides spanned 59% of SYN1, a synaptic vesicle-associated phosphoprotein previously implicated in ASD (Fig. 5b–c) [12].

### USIPP Antibodies Recognize Full-length Human SYNAPSIN Ia and the SYN1/USIPP Homologous Region

**Figure 6:**
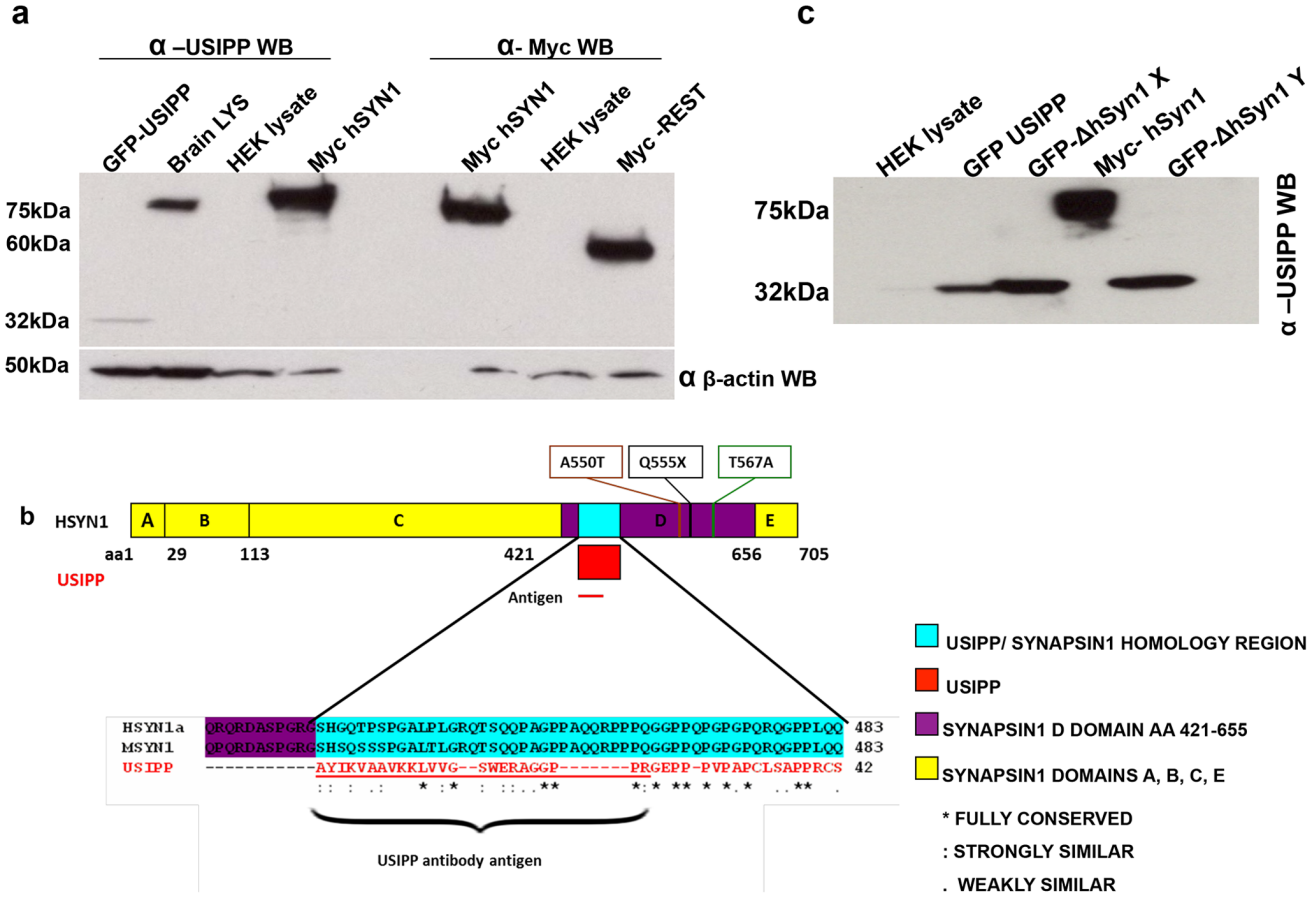
USIPP antibodies recognizes human SYNAPSIN 1A. (**a**) Myc-DDK- SYNAPSIN IA, Myc-REST, GFP-USIPP plasmids were transfected into HEK293 cells, incubated for 48 hours and lysed in NET-100 buffer. The lysates together with appropriate controls were resolved by SDS-PAGE and subjected to Western blot analysis with anti-USIPP or anti-Myc antibodies. (**b**) USIPP/SYN1 homology region, SYN1 D-DOMAIN and mutations implicated in autism and epilepsy. Multiple protein sequence alignment of human SYN1, murine SYN1 and USIPP with ClustalW shows a 31% USIPP identity with human and murine Synapsin1 from aa 431 to 483 in the D-domain; mutations shown in this domain have been implicated in autism and epilepsy. Antigen used for generating the anti-USIPP antibody is underlined in illustration. (**c**) USIPP antibody recognizes human Synapsin1a/USIPP homology region. USIPP/Syn1 homology region (aa 431 to 483 of SYNAPSIN I; eGFP-ΔhSyn1) was GFP-tagged and cloned into the pcDNA3.1 vector. Lysates from HEK293 cells transfected with Clones of eGFP-ΔhSyn1, (X & Y), GFP-USIPP or full-length Myc-hSyn1 were resolved with SDS-PAGE and subjected to anti-USIPP Western blot analysis.

To further evaluate the mass spectrometry results, full-length human SYNAPSIN Ia was Western blotted with anti-USIPP antibodies. Anti-USIPP antibodies recognized endogenous murine SYN1, GFP-USIPP, and human Myc-DDK-SYN1a (Fig. 6a). Also, staining of mouse dentate gyrus with anti-Synapsin I and anti-USIPP showed overlapping staining patterns (Fig. S4A). Human and murine SYN1 are composed of domains A–E [40] which when aligned with USIPP show a region of 31% identity between residues 431 and 483 in the SYN1 D domain. Strikingly, this domain contains most of the SYN1 mutations (A550T, Q555X, and T567A) previously reported in ASD (Fig. 6b) [12]. Alignments suggested that the anti-USIPP antibodies might specifically recognize D-domain residues 431 to 483. To test this, the USIPP/Syn1 homology region (residues 431 to 483; eGFP-ΔhSYN1) was cloned into an expression vector and expressed in HEK293 cells; cell extracts were then immunoblotted with anti-USIPP. The antibodies recognized GFP-USIPP, full length SYN1 (Myc-DDK-Syn1), and two independent eGFP-ΔhSYN1 clones (X and Y) (Fig. 6c). Fig. 5c shows representative results of the amino acid Atomic Mass Units (AMUs) and mass to ion ratio chromatograms of a single peptide of SYN1, identified from the mass spectrometry. This peptide encompasses the entire region homologous to USIPP (residues 431 to 483). Taken together, these findings demonstrate that USIPP and SYN1 share a structural epitope that physically interacts with PRICKLE1.

#### Endogenous SYN co-localizes with PRICKLE in the mouse brain and Drosophila neuromuscular junction, and coimmunoprecipitates with PRICKLE1 in mouse brain

**Figure 7:**
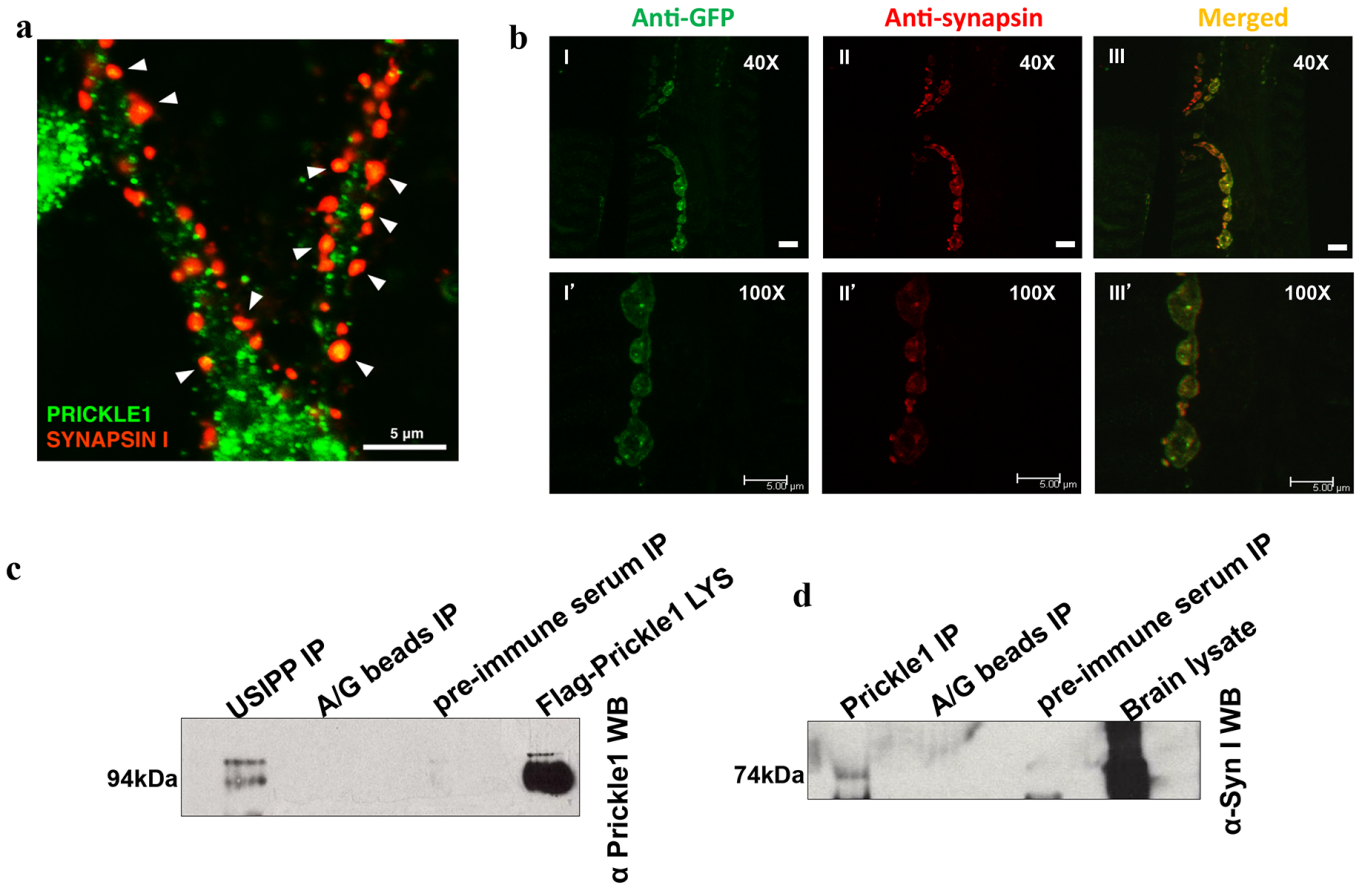
Endogenous SYN co-localizes with PRICKLE in the mouse brain and *Drosophila* neuromuscular junction, and coimmunoprecipitates with PRICKLE1 in mouse brain. (**a**) Co-localization of Prickle1 with Synapsin I in mouse hippocampal neurons in primary culture. Merged fluorescence image shows endogenous expression of PRICKLE1 (green) and SYNAPSIN I (red). Hippocampal neurons were fixed 10 days post-culture and immunostained with PRICKLE1 and SYNAPSIN I primary antibodies followed by AlexaFluor568 (Synapsin1) and AlexaFluor488 (Prickle1) secondary antibodies (**b**) EGFP-Pk and Synapsin co-localize at the neuromuscular junction (NMJ) in *Drosophila* third instar larvae. (I–III) 40X or (I′–III′) 100X immunohistochemistry confocal images of EGFP-Pk (I and I′) and Synapsin (II and II′) visualized at synaptic boutons of larval NMJs, along with the relevant merged images (III and III′). Note that there is a substantial co-localization of the EGFP-Pk and Synapsin signals. Scale bars = 5 µm (40X) and 5 µm (100X). Anti-GFP = rabbit anti-GFP Anti-Synapsin = mouse anti-Synapsin. (**c**) Lysates prepared from wild-type mouse brain was incubated with A/G agarose beads and USIPP antibodies, pre-immune serum or no serum overnight. Immunoprecipitates eluted in Laemmli buffer, resolved by SDS-PAGE and subjected to Western blot analyses with anti-PRICKLE1. (**d**) Lysates prepared from wild-type mouse brain was incubated with A/G agarose beads and Prickle1 antibodies, pre-immune serum or no serum overnight. Immunoprecipitates eluted in Laemmli buffer, resolved by SDS-PAGE and subjected to Western blot analyses with anti-Synapsin I.

Immunostaining of cultured hippocampal neurons with anti-PRICKLE1 and anti-SYN1 demonstrated Prickle1 co-localizes with Syn1 (Fig. 7a). Moreover, Syn1 and Prickle1 co-immunoprecipitated from wild-type mouse brain lysates (Fig. 7c and 7d), demonstrating that the proteins physically interact *in vivo*. Since a role for the Prickle proteins in regulating seizures has been conserved throughout evolution (leading to seizures in *Drosophila*, zebrafish, mice and humans [24], [41]), we next tested if *Synapsin* homozygous mutant flies were seizure-prone when compared to Oregon-R control flies using the bang sensitivity assay we employed previously for *prickle* mutant flies [24]. In addition to the observation that the *Synapsin* flies are seizure-prone and take longer to recover from their seizures when compared to controls (Figure S5), the seizure activity was strikingly similar to that observed for the seizure-prone *prickle* flies [24], including hyperactivity resulting in flies flipping on their backs. These data indicate that *prickle* and *Synapsin* might be working in the same pathway, and prompted us to determine whether Prickle and Synapsin might physically interact in fruit flies. We co-stained *Drosophila* third instar larvae that express a GFP-tagged form of Prickle in all neurons (EGFP-Pk) with both anti-GFP and anti-Synapsin antibodies and observed co-localization of staining throughout larval neurons and terminal boutons of the neuromuscular junction (Fig. 7b). Thus, the physical interaction between Prickle and Synapsin is conserved through evolution.

#### Generation of PC12 inducible lines overexpressing wild-type and mutant PRICKLE1

**Figure 8:**
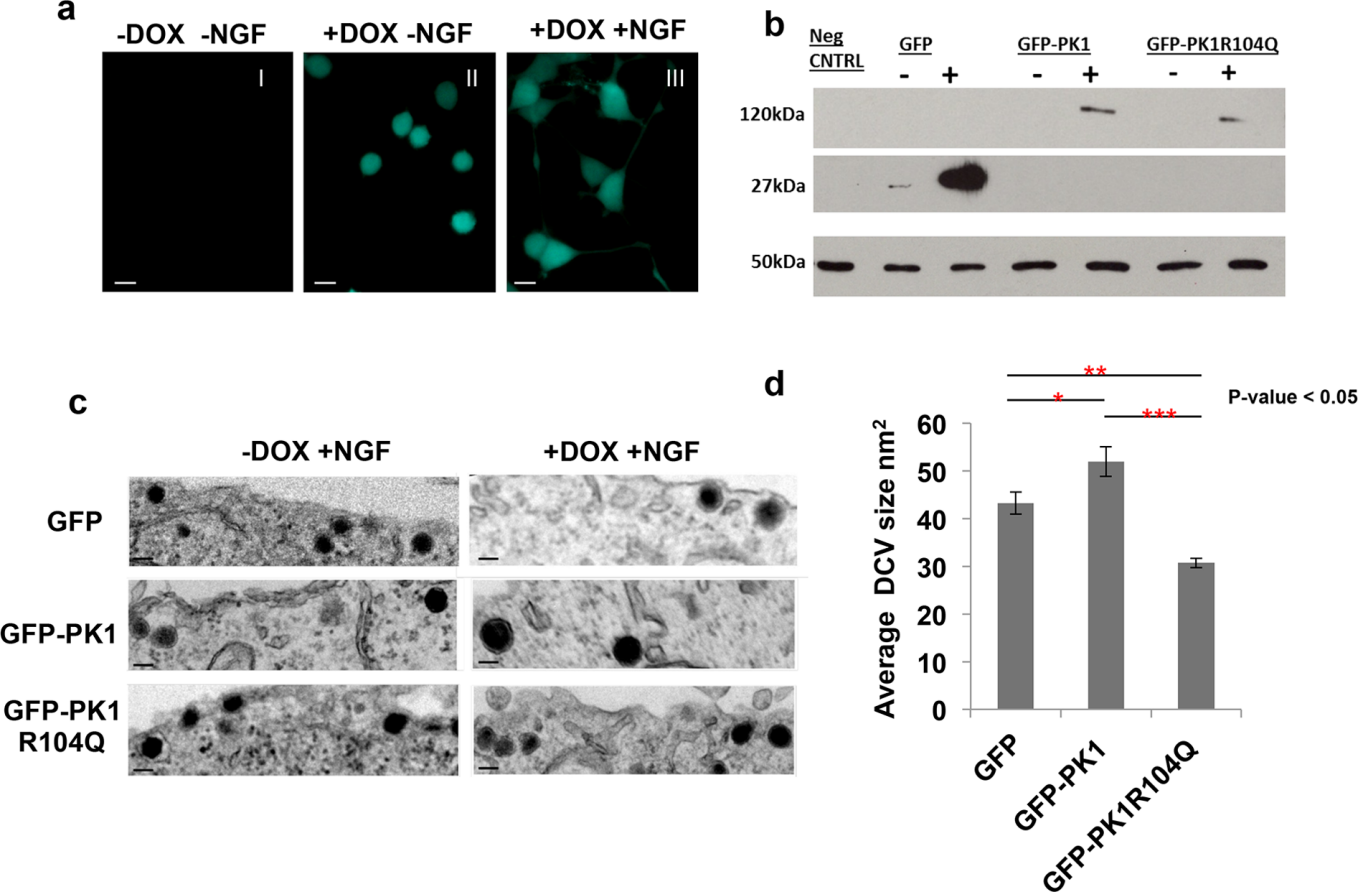
Mutant Prickle1 exhibits altered activity in inducible stable PC12 cells. (**a**) Fluorescence microscopy shows doxycycline (dox) induction of GFP in PC12 (Panel II) and differentiation in the presence of NGF after a 72-hr incubation period (Panel III). The size bars correspond to 20 nm. (**b**) Anti-GFP Western blot shows expression of stably transfected PC612 cells expressing GFP, GFP-PK1 or GFP-PK1R104Q under the control of dox-regulatable promoters. Lysates from dox-treated and untreated cell lines were resolved by SDS-PAGE and subjected to anti-GFP Western blot analysis. Image shows dox induction of transgenes. Anti-β actin Western Blot served as the loading control. (**c**) Transmission Electron Microscope (TEM) images of differentiated dox-treated and untreated PC12 cells, expressing GFP, WT, and mutant PRICKLE1, showing ultrastructure of Dense Core Vesicles (DCVs) in cytoplasm. The size bars correspond to 300 nm. Cell lines were differentiated with Nerve Growth Factor (NGF) with or without dox, for 7 days. (**d**) Using ImageJ, the average surface areas of DVCs in differentiated dox-treated were calculated to assess the effect of GFP, GFP-PK1 or GFP-PK1R104Q. Compared to the GFP control (43.22+/−13.78 nm^2^), wild-type PRICKLE1 significantly increased vesicle size (51.94+/−23.93 nm^2^, *p-value = 0.037) whereas mutant Prickle1 significantly decreased vesicle size (30.69+/−9.21 nm^2^,**p-value<0.005). The difference in activity between the wild-type and mutant Prickle1 was significant (***p-value<0.005).

PC6-3 cells are a subclone of rat pheochromocytoma PC12 cells that differentiate into a sympathetic neuron-like phenotype when treated with Nerve Growth Factor (NGF) [26]. Stably transfected PC12 clonal lines were isolated that, when exposed to doxycycline (a tetracycline derivate) inducibly expressed GFP, GFP-PRICKLE1, or mutant GFP-PRICKLE1R104Q (a mutation found in a large epilepsy pedigree [7]). Figure 8a shows a representative PC12 clonal line induced to express GFP (panel II) and the appearance of neurites after 72-hr incubation in NGF (panel III). The anti-GFP immunoblot in Figure 8b shows doxycycline induction of the GFP control, and GFP-tagged PRICKLE1 and mutant PRICKLE1.

#### Size of Dense-Core Vesicles (DCV) in PC12 cells increases with wild-type but not mutant PRICKLE1

SYN1 is a known synaptic protein, and SYN1-null neurons are defective in vesicle pool size and trafficking [12]. To examine whether PRICKLE1 also influences vesicle pool size and trafficking, we examined the effect of overexpressing the protein in PC12 cells–a model system for investigating neuronal differentiation in cultured cells [26]. Previous ASD studies have also employed PC12 cells as a model system [42], [43]. When PC12 cells are differentiated with nerve growth factor (NGF), they exhibit neural features, connect through synapse-like structures, and develop characteristics such as neurites, neurosecretion, and dense-core vesicles (DCVs) [44]. DCVs are secretory vesicles that contain neurotransmitters (e.g., norepinephrine and dopamine); and DCV size can be affected by various manipulations [45]. For example, overexpression of *REST* (RE-1 Silencing Transcription Factor) causes DCV size to decrease [29]. To investigate whether PRICKLE1 might be implicated in regulating vesicle size, PC12 cells expressing either the wild-type or mutant PRICKLE1 protein (with a R104Q encoding mutation from the first family described with PRICKLE1-related epilepsy [7]) were evaluated by transmission electron microscopy. This analysis revealed that the DCV ultrastructure was affected (Fig. 8c). Compared to GFP controls, overexpression of wild-type PRICKLE1 increased the average DCV size (p-value = 0.037, Fig. 8c–8d). Compared to the GFP controls however, cells overexpressing mutant PRICKLE1 (GFP-PRICKLE1R104Q) contained DCVs that were significantly smaller (p-value<0.005). Activity between the wild-type protein and mutant were highly significantly different (p-value<0.005). Despite the striking difference in size observed in DCVs between WT and mutant Prickle1, Synapsin I localization and expression remained unchanged in PC12 cells expressing GFP, GFP-Prickle1 or GFP-Prickle1R104Q (Figure S4B I–III).

## Discussion

Before being linked to ASDs, *PRICKLE1* was implicated in epilepsy [7], [24], [46]. Only later was *PRICKLE1* implicated in ASDs by the finding of rare, human *PRICKLE1* variants, and results from *Prickle1^+/−^* mice in behavioral studies showing their abnormal social behaviors, repetitive behaviors, and abnormal circadian rhythm, all consistent with an ASD phenotype. Like *PRICKLE1*, *SYN1* and *CNTNAP2* were also initially identified as epilepsy genes, but later studies associated them with ASDs [12], [47]–[49]. Compared to wild-type mice, mice heterozygous for a *PRICKLE1* mutation are also more active during the dark phase (the active phase for nocturnal animals) but less active during the rest phase, paralleling the perturbations in sleep and circadian rhythm characteristic of ASD individuals, and mouse and rat ASD models [34], [35]. Abnormalities in the circadian rhythm have been shown to be accompanied by increased serotonin in the frontal cortex in a rat model of autism [35]. Interestingly, an increase in serotonin neuron numbers were reported in postmortem brains of young autistic patients [50]. All of these behavioral data suggest a link between ASDs, perturbed circadian rhythms and epilepsy. The question remains: how are these genes linked at the functional level?

ASDs have been linked to mutations in the synaptic genes *NLGN3*, *NLGN4*, and *NRXN1*, as well as a postsynaptic scaffolding protein, *SHANK3* [51]. These genes form a complex that is crucial for both maintaining synaptic function, and balancing neuronal inhibition and excitation. A disruption in this balance is characteristic of ASDs, and might manifest in *Syn1*-null as repetitive behaviors [52], [53]. Reports that genes balancing synapse function also control circadian rhythm may suggest why sleep perturbation and ASD are linked [54]. Our findings support the idea that they are indeed linked, since *PRICKLE1* mutations appear to affect synaptic vesicles.

In addition to our experimental findings, this study also reports a novel methodology for isolating binding partners through epitope screening. Although the Y2H did not directly isolate a SYN1 peptide, we found peptide homology analysis and epitope screens may reveal bonafide interaction partners that might otherwise be considered false positives based on the nucleotide sequence alone. The experiments immunoprecipitating SYN1/PRICKLE1 complexes from brain extracts confirm that these proteins physically interact *in vivo.* Thus, screening peptide epitopes rather than just the nucleotide sequences encoding proteins might extend the usefulness of the Y2H screens.

The interaction we detected mapped to the D domain of SYN1 and the PET/LIM domain of PRICKLE1. This is particularly compelling because SYN1 mutations associated with epilepsy and ASDs are concentrated in the D domain, a region that physically binds to vesicles; mutations in this region impair vesicle pool size and trafficking [12], [55]. The observations that *SYN1* and *PRICKLE1* mutations are associated with epilepsy in humans and mice [7], [24], [53], [55] suggest they are both associated with the pathogenesis of the disease. Furthermore, SYN1 and PRICKLE1 physically interact, suggesting they may be involved in the same pathway.

Since *SYN1* mutations compromise synaptic vesicle size and trafficking, synaptic homeostasis may also be compromised in *PRICKLE1* mutants. It is therefore likely that PRICKLE1 functions at the synapse. Our hippocampal immunohistochemistry supports this idea, showing SYN1 colocalized with PSD-95, a synaptic protein also implicated in ASDs [56], [57]. Since PRICKLE1 is important for neurite outgrowth [58], [59] and SYN1 is important for neuronal development and synaptogenesis [40], it is possible that the genes function together to ensure normal neural connectivity.

Perhaps epilepsy and ASDs are both the result of compromised neural connectivity. It is quite intriguing that similar behaviors, including repetitive behaviors, have also been observed in Syn1 mutant mice [52], further suggesting that the genes share a pathway in common. Studies in PC12 cells show that DCV release of neurotransmitters mimics the connectivity process in neurons [45]. Wild-type PRICKLE1 increased DCV size but the mutant caused a decrease in size. This could suggest a reduction in neurotransmitters and a resultant change in neurotransmission. These findings further support a model in which PRICKLE1 may modulate synaptic vesicles in a SYN1-dependent manner. Disrupting this mechanism might affect synaptic homeostasis and contribute to the ASD phenotype. This and future studies would clarify ASD etiology and identify new therapeutic targets for intervention. In totality, this study links *PRICKLE1* to ASDs in both humans and mice and presents evidence that implicates PRICKLE1 in synaptic homeostasis.

## Supporting Information

Figure S1 ***Prickle1^+/−^*** **mice have normal body weight and temperature, and exhibit normal nociception, grip strength, and activity levels. A, B)** With p-values of 0.400 and 0.8445 for body weight and body temperature respectively, there were no significant differences between the wildtype controls (*n* = 24) and mutants (*n* = 16). **C) Mutant mice have normal grip strength.** A grip strength meter was used to assess the forelimb strength in control (*n* = 24) and mutant mice (*n* = 16). No significant difference was found between the genotypes, p-value = 0.1631. **D) Mutant mice have normal balance and wire hang strength.** A wire hang apparatus was used to measure balance and grip strength in controls (*n* = 24) and *Prickle1^+/−^* mice (*n* = 16), there was no significant difference between the groups (p-value = 0.2817). **E**) ***Prickle1^+/−^*** **mice display normal nociception.** The Hot plate test was used to measure response to painful stimuli in the control (*n* = 24) and mutant mice (*n* = 16). Here, latency to the first paw response was recorded. The paw response was a foot shake, a paw lick, or lifting both forepaws simultaneously. P-value = 0.426. **F) Mutant mice display similar level of activity with controls.** Activity was measured by assessing locomotor activity with the open field test. There was no significant difference between the controls and mutants during the day or at night. Both genotypes exhibited similar levels of activity. Night p-value = 0.367, day p-value = 0.8298.
(TIF)

Figure S2 **Wildtype controls and** ***Prickle1^+/−^*** **mutant mice display normal response to 2,3,5-Trimethyl-3-thiazoline (TMT) odor and normal context fear conditioning**. **A)** *Prickle1^+/−^* mice froze in fear in response to TMT like the controls. There was no significant difference between the genotypes. P-value = 0.968 **B)** ***Prickle1^+/−^*** **mice display normal context fear conditioning.** *Prickle1^+/−^* mice displayed similar normal context fear conditioning to the controls during training. There was no significant difference between the groups. P-value = 0.884.
(TIF)

Figure S3 ***Prickle1^+/−^*** **mutant mice and controls display normal cued fear conditioning**. During the fear conditioning training (**A**) testing (**B**), no significant difference was found between the controls (*n* = 17) and *Prickle1^+/−^* mutants (*n* = 19). Training p-value = 0.774, Testing p-value = 0.942.
(TIF)

Figure S4 **Anti-USIPP and anti-Synapsin I antibodies show similar staining patterns in the dentate gyrus, and endogenous Synapsin I expression pattern in PC12 cells stably expressing WT or mutant Prickle1 are similar. A)** Anti-Synapsin I (Panel I/green) or anti-USIPP (Panel II/red) staining have similar patterns since both antibodies recognize Synapsin I in the mouse dentate gyrus (DG). Merged confocal immunofluorescent image of a single DG section (Panel III) shows co-labeling of anti-Synapsin I and anti-USIPP. Scale bars correspond to 50 µm. **B)** The expression pattern of Synapsin I in cells expressing GFP (I), GFP-Prickle1 (II) or GFP-Prickle1R104Q (III) was similar. All cells displayed a vesicular localization pattern. PC12 cells stably expressing GFP, GFP-Prickle1 or GFP-Prickle1R104Q under the control of doxycycline were differentiated with Nerve Growth Factor (NGF) at 100 ng/ml and doxycycline at 1.5 ug/ml for 48 hrs. Fixed cells were treated with rabbit anti-Synapsin I (red) mouse anti-Tubulin (grey) primary antibodies followed by goat anti-mouse 647 and goat anti-rabbit 568. Scale bars correspond to 20 µm.
(TIF)

Figure S5 ***Drosophila*** **flies that are homozygous for a loss-of-function** ***Synapsin*** **mutation** ***a*** **re predisposed to seizures**. Wild-type and *Syn^97^* homozygous mutant flies were subjected to the modified bang-sensitivity assay to measure seizure recovery time. *Syn^97^* homozygous mutant flies have significantly impaired seizure recovery for all time points when compared to same-aged control flies (Oregon-R). *p<1e-6.
(TIF)

File S1 **Contains the following tables: TABLE 1A: Top 100 Clones from Two-yeast Hybrid- Human PRICKLE1 vs Fetal Brain.** The top 100 sequenced clones from human PRICKLE1 vs human fetal brain cDNA Y2H with the highest confidence scores. **TABLE 1B : Top 100 Clones from Two-yeast Hybrid- Human PRICKLE1 vs Adult Brain.** The top 100 sequenced clones from human PRICKLE1 vs human adult brain cDNA Y2H with the highest confidence scores. **TABLE 2: 230 USIPP PSI BLAST Brain Expressed Proteins.** USIPP sequence was blasted against all human sequences in PSI-blast and produced 1000 hits. The list was narrowed down to 230 brain-expressed proteins.
(PDF)

Video S1 ***Prickle1^+/−^*** **mice exhibit repetitive movements consistent with ASD-like behavior.** A *Prickle1^+/−^* mouse (left) versus a wild-type littermate (right). Note repeated jumping behaviour in *Prickle1^+/−^* mouse. This behaviour is seen sporadically in the *Prickle1^+/−^* mice.
(MOV)
