# Supplementary figures and images for: PRICKLE1 Interaction with SYNAPSIN I Reveals a Role in Autism Spectrum Disorders
Source: PLoS One. 2013 Dec 3;8(12):e80737. doi: 10.1371/journal.pone.0080737 (PMC3849077; doi:10.1371/journal.pone.0080737)

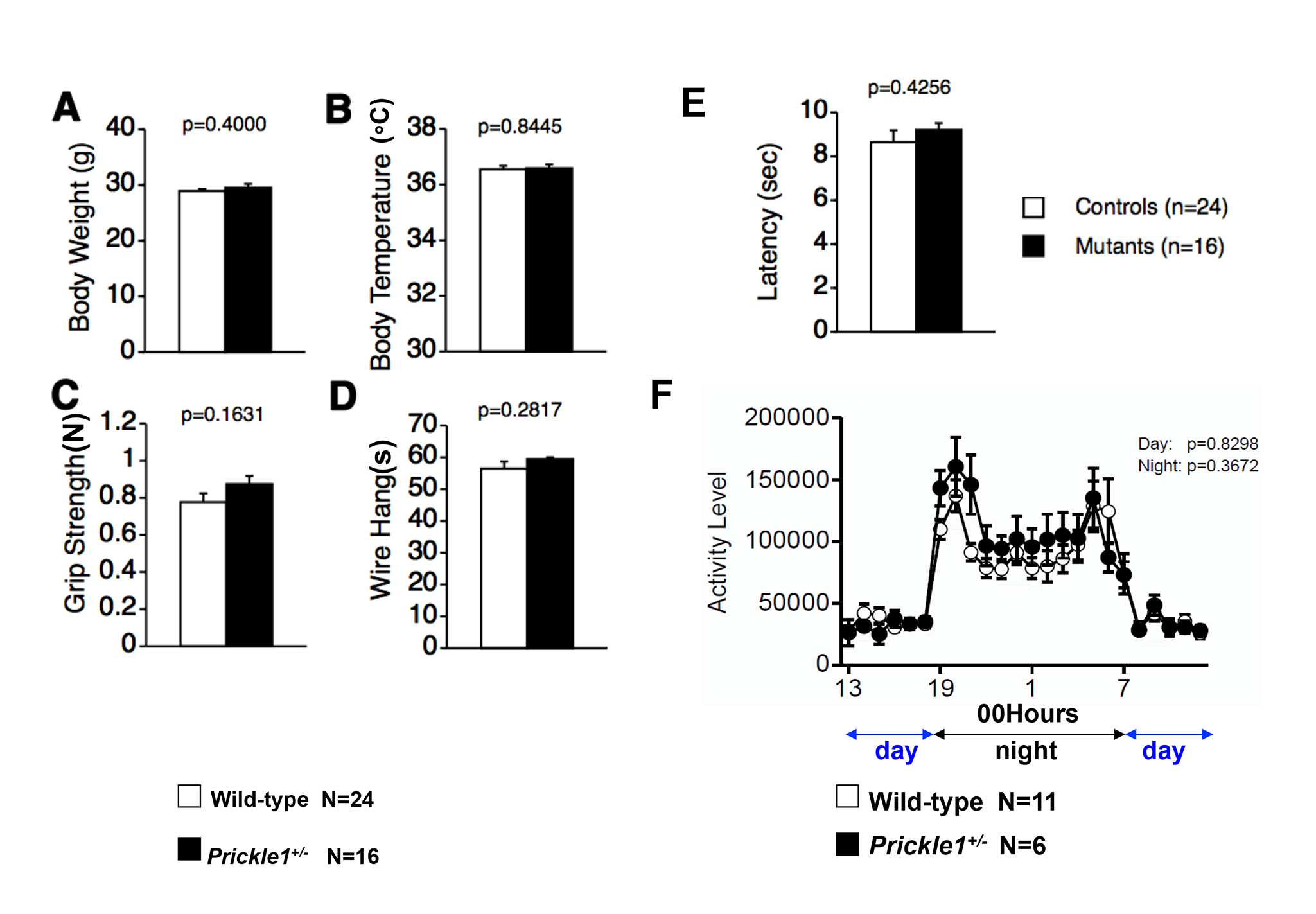

Supplement: Figure S1 — Prickle1+/− mice have normal body weight and temperature, and exhibit normal nociception, grip strength, and activity levels. A, B) With p-values of 0.400 and 0.8445 for body weight and body temperature respectively, there were no significant differences between the wildtype controls (n = 24) and mutants (n = 16). C) Mutant mice have normal grip strength. A grip strength meter was used to assess the forelimb strength in control (n = 24) and mutant mice (n = 16). No significant difference was found between the genotypes, p-value = 0.1631. D) Mutant mice have normal balance and wire hang strength. A wire hang apparatus was used to measure balance and grip strength in controls (n = 24) and Prickle1+/− mice (n = 16), there was no significant difference between the groups (p-value = 0.2817). E) Prickle1+/− mice display normal nociception. The Hot plate test was used to measure response to painful stimuli in the control (n = 24) and mutant mice (n = 16). Here, latency to the first paw response was recorded. The paw response was a foot shake, a paw lick, or lifting both forepaws simultaneously. P-value = 0.426. F) Mutant mice display similar level of activity with controls. Activity was measured by assessing locomotor activity with the open field test. There was no significant difference between the controls and mutants during the day or at night. Both genotypes exhibited similar levels of activity. Night p-value = 0.367, day p-value = 0.8298. (TIF) [file pone.0080737.s001.tif]

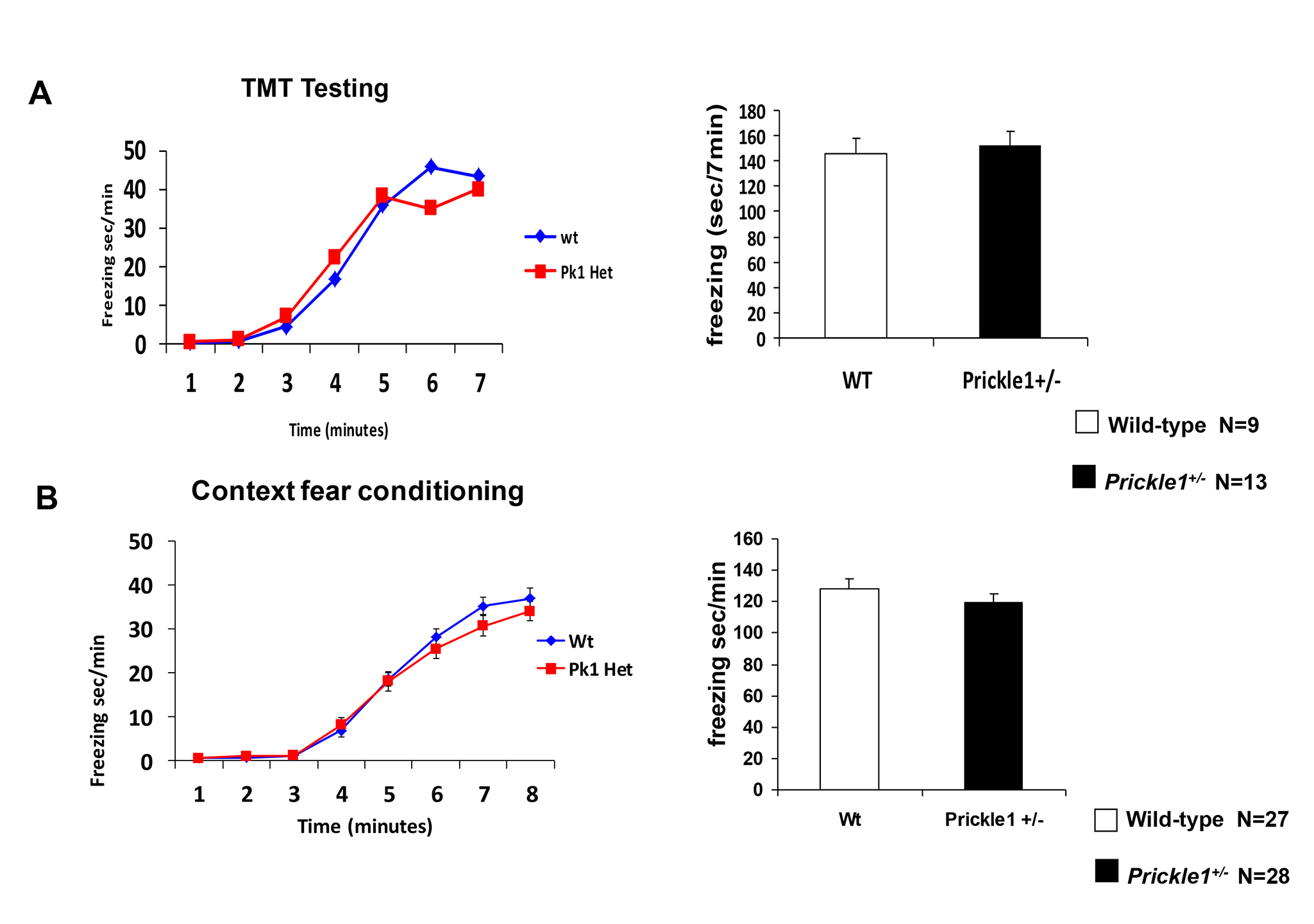

Supplement: Figure S2 — Wildtype controls and Prickle1+/− mutant mice display normal response to 2,3,5-Trimethyl-3-thiazoline (TMT) odor and normal context fear conditioning. A) Prickle1+/− mice froze in fear in response to TMT like the controls. There was no significant difference between the genotypes. P-value = 0.968 B) Prickle1+/− mice display normal context fear conditioning. Prickle1+/− mice displayed similar normal context fear conditioning to the controls during training. There was no significant difference between the groups. P-value = 0.884. (TIF) [file pone.0080737.s002.tif]

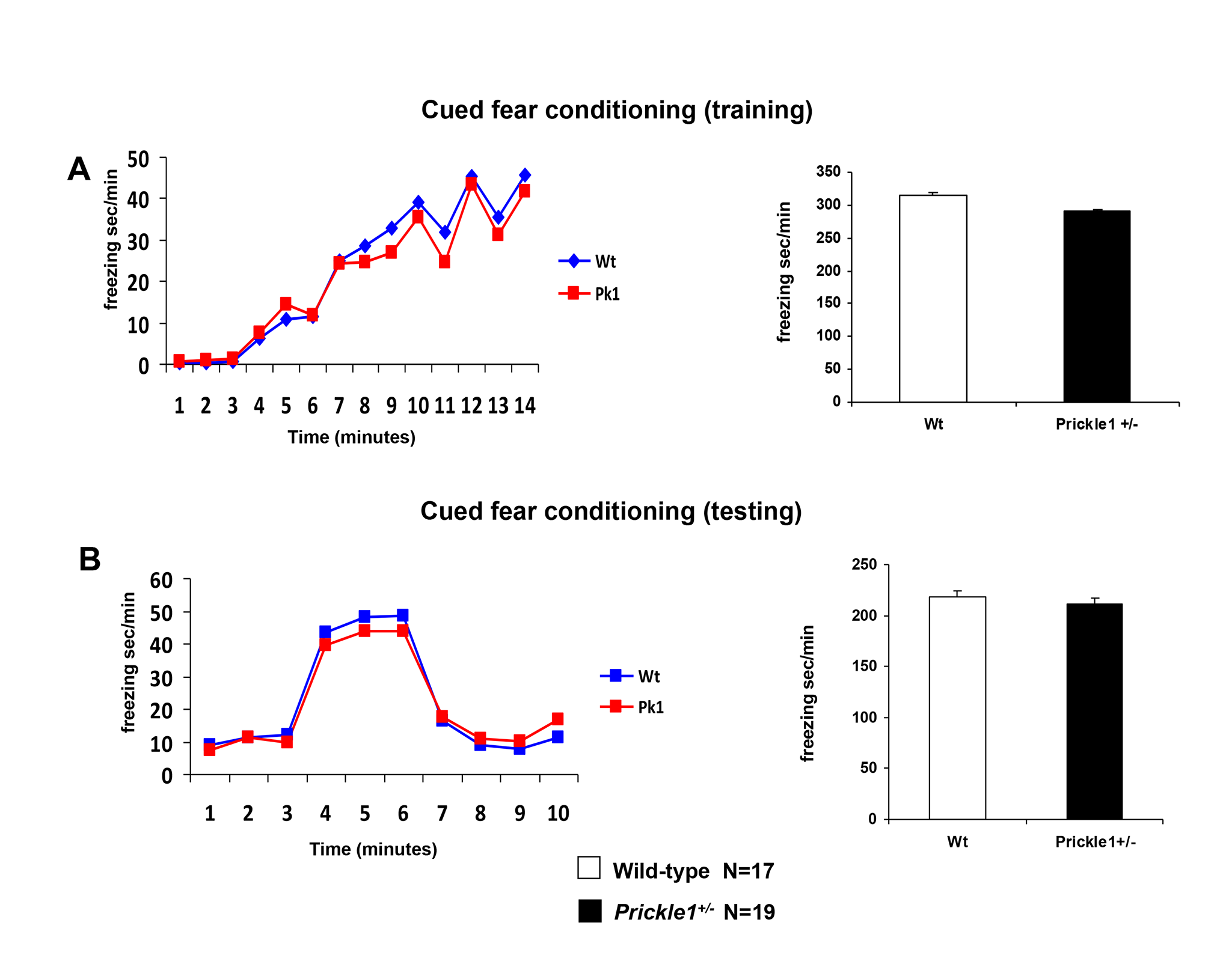

Supplement: Figure S3 — Prickle1+/− mutant mice and controls display normal cued fear conditioning. During the fear conditioning training (A) testing (B), no significant difference was found between the controls (n = 17) and Prickle1+/− mutants (n = 19). Training p-value = 0.774, Testing p-value = 0.942. (TIF) [file pone.0080737.s003.tif]

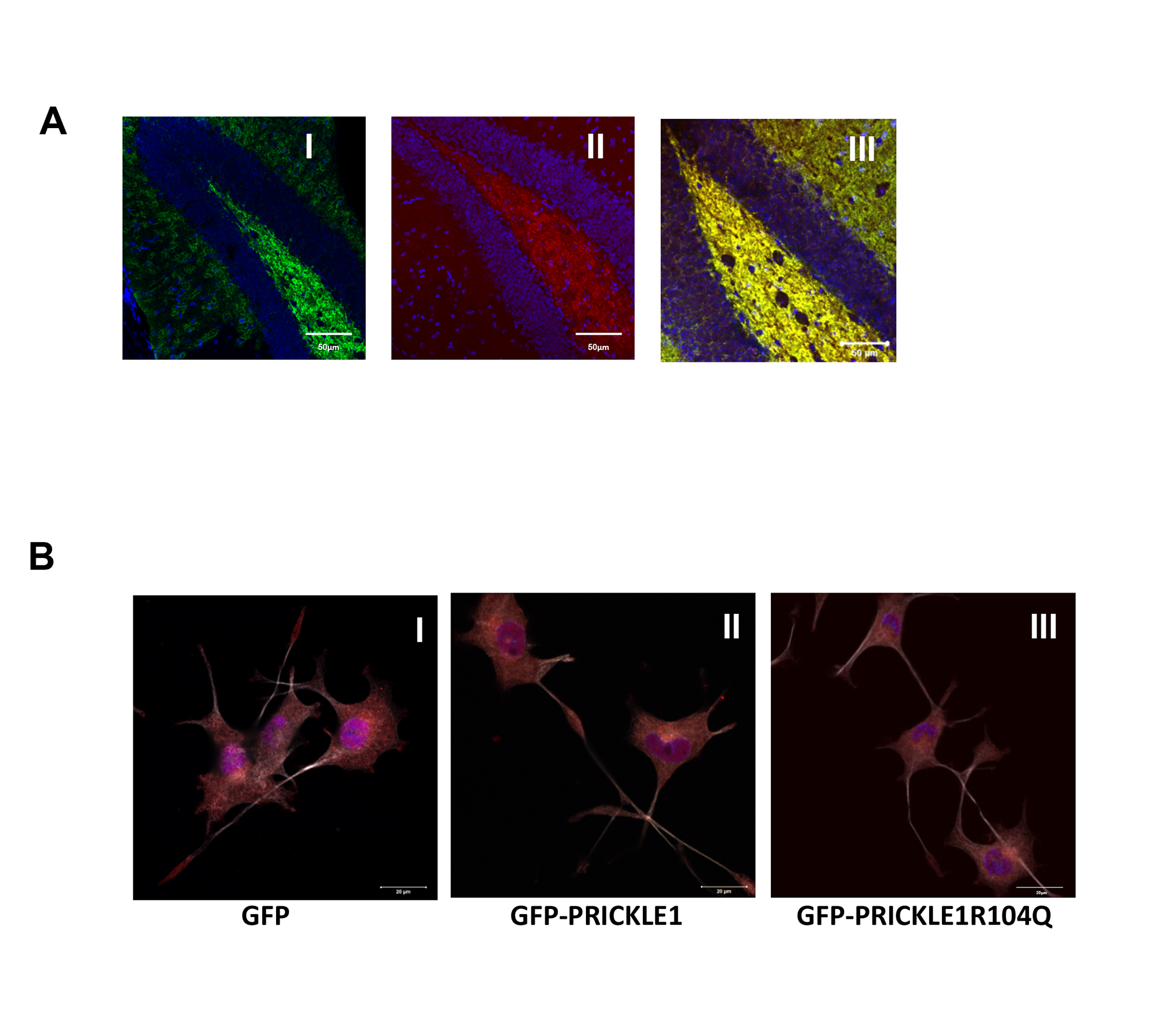

Supplement: Figure S4 — Anti-USIPP and anti-Synapsin I antibodies show similar staining patterns in the dentate gyrus, and endogenous Synapsin I expression pattern in PC12 cells stably expressing WT or mutant Prickle1 are similar. A) Anti-Synapsin I (Panel I/green) or anti-USIPP (Panel II/red) staining have similar patterns since both antibodies recognize Synapsin I in the mouse dentate gyrus (DG). Merged confocal immunofluorescent image of a single DG section (Panel III) shows co-labeling of anti-Synapsin I and anti-USIPP. Scale bars correspond to 50 µm. B) The expression pattern of Synapsin I in cells expressing GFP (I), GFP-Prickle1 (II) or GFP-Prickle1R104Q (III) was similar. All cells displayed a vesicular localization pattern. PC12 cells stably expressing GFP, GFP-Prickle1 or GFP-Prickle1R104Q under the control of doxycycline were differentiated with Nerve Growth Factor (NGF) at 100 ng/ml and doxycycline at 1.5 ug/ml for 48 hrs. Fixed cells were treated with rabbit anti-Synapsin I (red) mouse anti-Tubulin (grey) primary antibodies followed by goat anti-mouse 647 and goat anti-rabbit 568. Scale bars correspond to 20 µm. (TIF) [file pone.0080737.s004.tif]

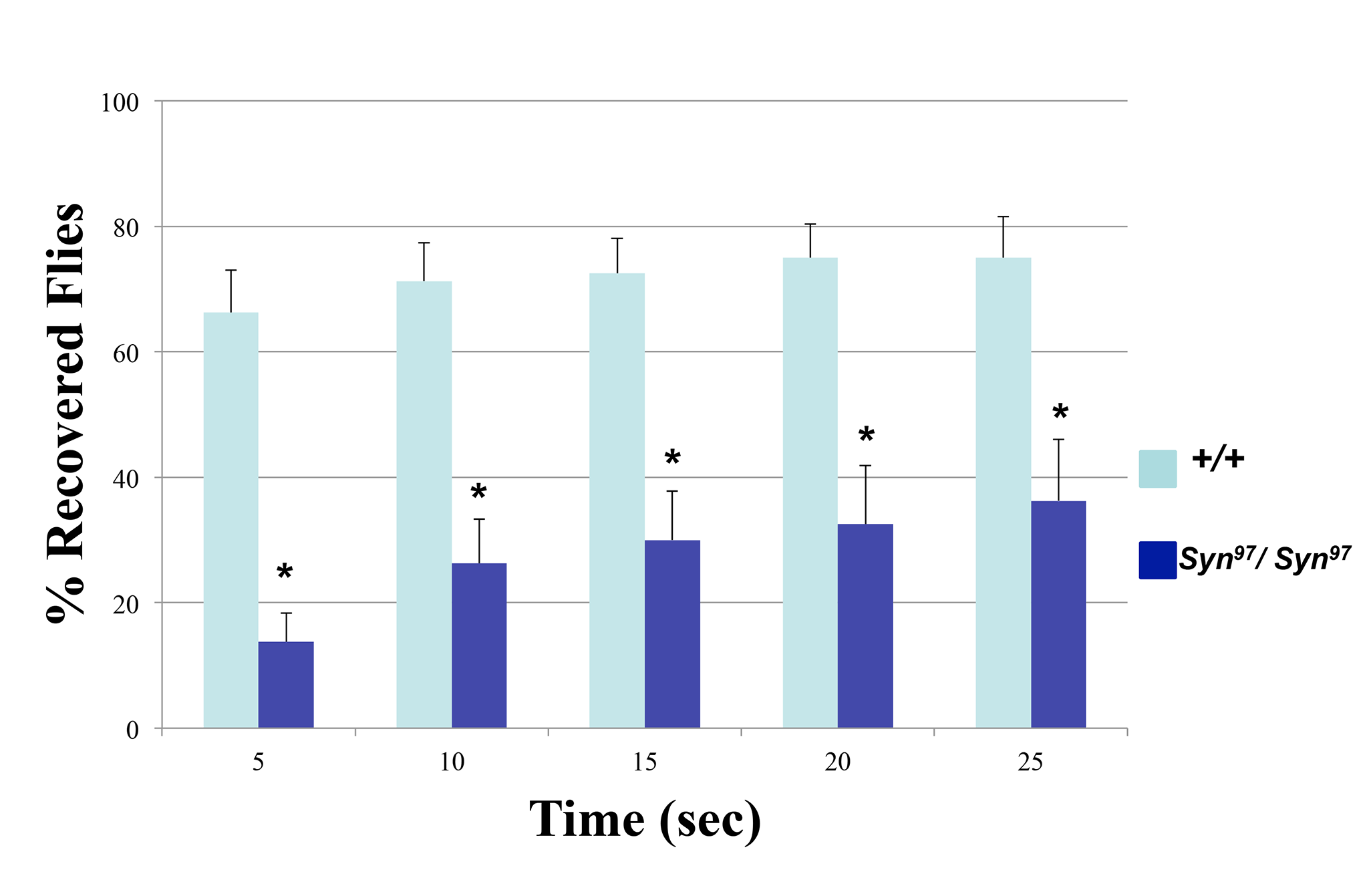

Supplement: Figure S5 — Drosophila flies that are homozygous for a loss-of-function Synapsin mutation a re predisposed to seizures. Wild-type and Syn97 homozygous mutant flies were subjected to the modified bang-sensitivity assay to measure seizure recovery time. Syn97 homozygous mutant flies have significantly impaired seizure recovery for all time points when compared to same-aged control flies (Oregon-R). *p<1e-6. (TIF) [file pone.0080737.s005.tif]
