# Supplementary material for: PRICKLE1 Interaction with SYNAPSIN I Reveals a Role in Autism Spectrum Disorders
Source: PLoS One. 2013 Dec 3;8(12):e80737. doi: 10.1371/journal.pone.0080737 (PMC3849077; doi:10.1371/journal.pone.0080737)
Supplement: File S1 — Contains the following tables: TABLE 1A: Top 100 Clones from Two-yeast Hybrid- Human PRICKLE1 vs Fetal Brain. The top 100 sequenced clones from human PRICKLE1 vs human fetal brain cDNA Y2H with the highest confidence scores. TABLE 1B : Top 100 Clones from Two-yeast Hybrid- Human PRICKLE1 vs Adult Brain. The top 100 sequenced clones from human PRICKLE1 vs human adult brain cDNA Y2H with the highest confidence scores. TABLE 2: 230 USIPP PSI BLAST Brain Expressed Proteins. USIPP sequence was blasted against all human sequences in PSI-blast and produced 1000 hits. The list was narrowed down to 230 brain-expressed proteins. (PDF) [file pone.0080737.s006.pdf]

SUPPORTING TABLES

TABLE 1A: TOP 100 CLONES FROM YEAST TWO-HYBRID- HUMAN PRICKLE VS HUMAN FETAL BRAIN

	Clone Name	Global PBS	Gene Notes	% Id 5p/3p
**1**	HFBR_RP1_hgx1866v1_pB27_C-60	A	18S ribosomal RNA	86.9 / 73.1
**3**	HFBR_RP1_hgx1866v1_pB27_C-85	A	18S ribosomal RNA	85.4 / 80.6
**4**	HFBR_RP1_hgx1866v1_pB27_C-7	A	18S ribosomal RNA	85.0 / 85.0
**5**	HFBR_RP1_hgx1866v1_pB27_D-19	A	18S ribosomal RNA	100.0 / 100.0
**6**	HFBR_RP1_hgx1866v1_pB27_D-8	A	18S ribosomal RNA	100.0 / 100.0
**7**	HFBR_RP1_hgx1866v1_pB27_C-50	A	18S ribosomal RNA	91.1 / 79.4
**8**	HFBR_RP1_hgx1866v1_pB27_D-93	A	18S ribosomal RNA	99.8 / 98.1
**9**	HFBR_RP1_hgx1866v1_pB27_D-58	A	18S ribosomal RNA	99.8 / 98.8
**10**	HFBR_RP1_hgx1866v1_pB27_D-74	A	18S ribosomal RNA	100.0 / 100.0
**11**	HFBR_RP1_hgx1866v1_pB27_C-34	A	18S ribosomal RNA	85.3 / 93.8
**12**	HFBR_RP1_hgx1866v1_pB27_D-6	A	18S ribosomal RNA	100.0 / 100.0
**13**	HFBR_RP1_hgx1866v1_pB27_C-74	A	18S ribosomal RNA	91.8 / 76.1
**14**	HFBR_RP1_hgx1866v1_pB27_C-61	A	18S ribosomal RNA	91.9 / 78.1
**15**	HFBR_RP1_hgx1866v1_pB27_C-47	A	18S ribosomal RNA	89.3 / 79.3
**16**	HFBR_RP1_hgx1866v1_pB27_D-23	A	18S ribosomal RNA	100.0 / 100.0
**17**	HFBR_RP1_hgx1866v1_pB27_D-31	A	18S ribosomal RNA	100.0 / 100.0
**18**	HFBR_RP1_hgx1866v1_pB27_D-17	A	18S ribosomal RNA	100.0 / 95.2
**19**	HFBR_RP1_hgx1866v1_pB27_D-38	A	18S ribosomal RNA	100.0 / 100.0
**20**	HFBR_RP1_hgx1866v1_pB27_D-53	A	18S ribosomal RNA	99.8 / 99.8
**21**	HFBR_RP1_hgx1866v1_pB27_C-53	A	18S ribosomal RNA	88.6 / 85.1
**22**	HFBR_RP1_hgx1866v1_pB27_C-12	A	18S ribosomal RNA	84.8 / 78.0
**23**	HFBR_RP1_hgx1866v1_pB27_D-67	A	18S ribosomal RNA	100.0 / 100.0
**24**	HFBR_RP1_hgx1866v1_pB27_D-89	A	18S ribosomal RNA	100.0 / 99.8
**25**	HFBR_RP1_hgx1866v1_pB27_C-5	A	18S ribosomal RNA	83.8 / 84.3
**26**	HFBR_RP1_hgx1866v1_pB27_C-80	A	18S ribosomal RNA	84.7 / 81.1
**27**	HFBR_RP1_hgx1866v1_pB27_C-8	A	18S ribosomal RNA	91.6 / 80.4
**28**	HFBR_RP1_hgx1866v1_pB27_D-52	A	18S ribosomal RNA	100.0 / 100.0
**29**	HFBR_RP1_hgx1866v1_pB27_D-46	A	18S ribosomal RNA	99.8 / 99.8
**30**	HFBR_RP1_hgx1866v1_pB27_C-11	A	18S ribosomal RNA	82.9 / 72.1
**31**	HFBR_RP1_hgx1866v1_pB27_C-21	A	18S ribosomal RNA	87.6 / 80.5
**32**	HFBR_RP1_hgx1866v1_pB27_D-59	A	18S ribosomal RNA	100.0 / 99.8
**33**	HFBR_RP1_hgx1866v1_pB27_D-36	A	18S ribosomal RNA	100.0 / 100.0
**34**	HFBR_RP1_hgx1866v1_pB27_C-13	A	18S ribosomal RNA	82.0 / 88.0
**35**	HFBR_RP1_hgx1866v1_pB27_C-67	A	18S ribosomal RNA	78.7 / 78.0
**36**	HFBR_RP1_hgx1866v1_pB27_D-34	A	18S ribosomal RNA	100.0 / 100.0
**37**	HFBR_RP1_hgx1866v1_pB27_D-80	A	18S ribosomal RNA	100.0 / 100.0
**38**	HFBR_RP1_hgx1866v1_pB27_C-48	A	18S ribosomal RNA	79.8 / 82.8
**39**	HFBR_RP1_hgx1866v1_pB27_C-55	A	18S ribosomal RNA	84.7 / 89.5
**40**	HFBR_RP1_hgx1866v1_pB27_C-82	A	18S ribosomal RNA	86.0 / 79.5
**41**	HFBR_RP1_hgx1866v1_pB27_C-57	A	18S ribosomal RNA	79.9 / 83.1

**42**	HFBR_RP1_hgx1866v1_pB27_D-90	A	18S ribosomal RNA	100.0 / 99.8
**43**	HFBR_RP1_hgx1866v1_pB27_D-49	A	18S ribosomal RNA	100.0 / 100.0
**44**	HFBR_RP1_hgx1866v1_pB27_D-50	A	18S ribosomal RNA	100.0 / 99.1
**45**	HFBR_RP1_hgx1866v1_pB27_D-51	A	18S ribosomal RNA	100.0 / 99.8
**46**	HFBR_RP1_hgx1866v1_pB27_C-20	A	18S ribosomal RNA	90.0 / 94.1
**47**	HFBR_RP1_hgx1866v1_pB27_D-72	A	18S ribosomal RNA	100.0 / 98.9
**48**	HFBR_RP1_hgx1866v1_pB27_C-49	A	18S ribosomal RNA	85.1 / 81.6
**49**	HFBR_RP1_hgx1866v1_pB27_C-38	A	18S ribosomal RNA	90.9 / 89.2
**50**	HFBR_RP1_hgx1866v1_pB27_D-44	A	18S ribosomal RNA	99.4 / 99.4
**51**	HFBR_RP1_hgx1866v1_pB27_D-82	A	18S ribosomal RNA	99.6 / 99.4
**52**	HFBR_RP1_hgx1866v1_pB27_D-73	A	18S ribosomal RNA	100.0 / 98.7
**53**	HFBR_RP1_hgx1866v1_pB27_D-37	A	18S ribosomal RNA	100.0 / 100.0
**54**	HFBR_RP1_hgx1866v1_pB27_D-7	A	18S ribosomal RNA	100.0 / 99.8
**55**	HFBR_RP1_hgx1866v1_pB27_D-76	A	18S ribosomal RNA	94.2 / 85.8
**56**	HFBR_RP1_hgx1866v1_pB27_C-33	A	18S ribosomal RNA	87.1 / 96.5
**57**	HFBR_RP1_hgx1866v1_pB27_D-22	A	18S ribosomal RNA	100.0 / 100.0
**58**	HFBR_RP1_hgx1866v1_pB27_D-66	A	18S ribosomal RNA	100.0 / 100.0
**59**	HFBR_RP1_hgx1866v1_pB27_C-45	A	18S ribosomal RNA	69.2 / 79.3
**60**	HFBR_RP1_hgx1866v1_pB27_D-28	A	18S ribosomal RNA	88.5 / 96.7
**61**	HFBR_RP1_hgx1866v1_pB27_C-54	A	18S ribosomal RNA	86.1 / 80.8
**62**	HFBR_RP1_hgx1866v1_pB27_D-77	A	18S ribosomal RNA	100.0 / 98.8
**63**	HFBR_RP1_hgx1866v1_pB27_C-37	A	18S ribosomal RNA	78.6 / 89.2
**64**	HFBR_RP1_hgx1866v1_pB27_D-92	A	18S ribosomal RNA	100.0 / 100.0
**65**	HFBR_RP1_hgx1866v1_pB27_D-87	A	18S ribosomal RNA	100.0 / 99.5
**66**	HFBR_RP1_hgx1866v1_pB27_C-73	A	18S ribosomal RNA	82.5 / 68.5
**67**	HFBR_RP1_hgx1866v1_pB27_C-14	A	18S ribosomal RNA	88.6 / 94.4
**68**	HFBR_RP1_hgx1866v1_pB27_D-35	A	18S ribosomal RNA	100.0 / 100.0
**69**	HFBR_RP1_hgx1866v1_pB27_D-71	A	18S ribosomal RNA	100.0 / 99.5
**70**	HFBR_RP1_hgx1866v1_pB27_D-41	A	18S ribosomal RNA	100.0 / 100.0
**71**	HFBR_RP1_hgx1866v1_pB27_D-18	A	18S ribosomal RNA	100.0 / 100.0
**72**	HFBR_RP1_hgx1866v1_pB27_D-68	A	18S ribosomal RNA	100.0 / 100.0
**73**	HFBR_RP1_hgx1866v1_pB27_D-56	A	18S ribosomal RNA	100.0 / 100.0
**74**	HFBR_RP1_hgx1866v1_pB27_D-24	A	18S ribosomal RNA	100.0 / 100.0
**75**	HFBR_RP1_hgx1866v1_pB27_D-57	A	18S ribosomal RNA	96.3 / 94.5
**76**	HFBR_RP1_hgx1866v1_pB27_D-10	A	18S ribosomal RNA	100.0 / 100.0
**77**	HFBR_RP1_hgx1866v1_pB27_C-9	A	18S ribosomal RNA	74.3 / 91.3
**78**	HFBR_RP1_hgx1866v1_pB27_C-72	A	18S ribosomal RNA	76.6 / 96.2
**79**	HFBR_RP1_hgx1866v1_pB27_D-4	A	18S ribosomal RNA	100.0 / 100.0
**80**	HFBR_RP1_hgx1866v1_pB27_C-68	A	18S ribosomal RNA	70.2 / 82.0
**81**	HFBR_RP1_hgx1866v1_pB27_D-13	A	18S ribosomal RNA	100.0 / 99.8
**82**	HFBR_RP1_hgx1866v1_pB27_D-20	A	18S ribosomal RNA	100.0 / 100.0
**83**	HFBR_RP1_hgx1866v1_pB27_D-84	A	18S ribosomal RNA	100.0 / 100.0
**84**	HFBR_RP1_hgx1866v1_pB27_C-15	A	18S ribosomal RNA	83.1 / 83.7
**85**	HFBR_RP1_hgx1866v1_pB27_D-30	A	18S ribosomal RNA	100.0 / 99.6
**86**	HFBR_RP1_hgx1866v1_pB27_D-39	A	18S ribosomal RNA	100.0 / 99.7
**87**	HFBR_RP1_hgx1866v1_pB27_D-85	A	18S ribosomal RNA	99.6 / 100.0
**88**	HFBR_RP1_hgx1866v1_pB27_D-16	A	18S ribosomal RNA	100.0 / 100.0
**89**	HFBR_RP1_hgx1866v1_pB27_D-27	A	18S ribosomal RNA	100.0 / 100.0

**90**	HFBR_RP1_hgx1866v1_pB27_D-26	A	18S ribosomal RNA	100.0 / 100.0
**91**	HFBR_RP1_hgx1866v1_pB27_D-95	A	18S ribosomal RNA	100.0 / 100.0
**92**	HFBR_RP1_hgx1866v1_pB27_D-61	A	18S ribosomal RNA	100.0 / 100.0
**93**	HFBR_RP1_hgx1866v1_pB27_D-25	A	18S ribosomal RNA	100.0 / 100.0
**94**	HFBR_RP1_hgx1866v1_pB27_D-94	A	18S ribosomal RNA	100.0 / 100.0
**95**	HFBR_RP1_hgx1866v1_pB27_D-81	A	18S ribosomal RNA	100.0 / 100.0
**96**	HFBR_RP1_hgx1866v1_pB27_C-81	A	18S ribosomal RNA	78.3 / 81.8
**97**	HFBR_RP1_hgx1866v1_pB27_C-70	A	18S ribosomal RNA	87.3 / 95.3
**98**	HFBR_RP1_hgx1866v1_pB27_C-19	A	18S ribosomal RNA	87.5 / 79.8
**99**	HFBR_RP1_hgx1866v1_pB27_C-69	A	18S ribosomal RNA	82.4 / 97.0
**100**	HFBR_RP1_hgx1866v1_pB27_C-65	A	18S ribosomal RNA	86.8 / 86.0

TABLE 1B : TOP 100 CLONES YEAST TWO-HYBRID- HUMAN PRICKLE VS HUMAN ADULT BRAIN LIBRARY

	**Clone Name**	**Global PBS**	**Gene Notes**	**% Id 5p/3p**
**1**	HBR_RP1_hgx1866v1_pB27_B-344	A	18S ribosomal RNA	91.1
**2**	HBR_RP1_hgx1866v1_pB27_B-378	A	18S ribosomal RNA	97.7
**3**	HBR_RP1_hgx1866v1_pB27_B-292	A	18S ribosomal RNA	96.3
**4**	HBR_RP1_hgx1866v1_pB27_B-379	A	18S ribosomal RNA	89.8
**5**	HBR_RP1_hgx1866v1_pB27_B-201	A	18S ribosomal RNA	90.4
**6**	HBR_RP1_hgx1866v1_pB27_B-269	A	18S ribosomal RNA	97.2
**7**	HBR_RP1_hgx1866v1_pB27_B-257	A	18S ribosomal RNA	95.2
**8**	HBR_RP1_hgx1866v1_pB27_B-190	A	18S ribosomal RNA	94.3
**9**	HBR_RP1_hgx1866v1_pB27_B-304	A	18S ribosomal RNA	81.7
**10**	HBR_RP1_hgx1866v1_pB27_B-284	A	18S ribosomal RNA	86.5
**11**	HBR_RP1_hgx1866v1_pB27_B-11	A	18S ribosomal RNA	88.6
**12**	HBR_RP1_hgx1866v1_pB27_B-317	A	18S ribosomal RNA	96.7
**13**	HBR_RP1_hgx1866v1_pB27_B-368	A	18S ribosomal RNA	94.4
**14**	HBR_RP1_hgx1866v1_pB27_B-352	A	18S ribosomal RNA	93.5 / 93.8
**15**	HBR_RP1_hgx1866v1_pB27_B-309	A	18S ribosomal RNA	90.5 / 92.2
**16**	HBR_RP1_hgx1866v1_pB27_B-143	A	18S ribosomal RNA	91.1 / 91.7
**17**	HBR_RP1_hgx1866v1_pB27_B-161	A	18S ribosomal RNA	95.5 / 93.0
**18**	HBR_RP1_hgx1866v1_pB27_B-48	A	18S ribosomal RNA	94.9 / 85.2
**19**	HBR_RP1_hgx1866v1_pB27_B-277	A	18S ribosomal RNA	90.5 / 81.5
**20**	HBR_RP1_hgx1866v1_pB27_B-380	A	18S ribosomal RNA	92.2 / 79.5
**21**	HBR_RP1_hgx1866v1_pB27_B-285	A	18S ribosomal RNA	92.2 / 92.1
**22**	HBR_RP1_hgx1866v1_pB27_B-9	A	18S ribosomal RNA	97.7 / 55.4
**23**	HBR_RP1_hgx1866v1_pB27_B-165	A	18S ribosomal RNA	98.4 / 93.0
**24**	HBR_RP1_hgx1866v1_pB27_B-202	A	18S ribosomal RNA	93.2 / 89.8
**25**	HBR_RP1_hgx1866v1_pB27_B-203	A	18S ribosomal RNA	96.6 / 92.3
**26**	HBR_RP1_hgx1866v1_pB27_B-170	A	18S ribosomal RNA	94.4 / 93.3
**27**	HBR_RP1_hgx1866v1_pB27_B-273	A	18S ribosomal RNA	90.3 / 89.9
**28**	HBR_RP1_hgx1866v1_pB27_B-275	A	18S ribosomal RNA	95.4

**29**	HBR_RP1_hgx1866v1_pB27_B-272	A	18S ribosomal RNA	90.2 / 82.4
**30**	HBR_RP1_hgx1866v1_pB27_B-365	A	18S ribosomal RNA	100.0 / 99.3
**31**	HBR_RP1_hgx1866v1_pB27_B-164	A	18S ribosomal RNA	100.0 / 100.0
**32**	HBR_RP1_hgx1866v1_pB27_B-177	A	18S ribosomal RNA	99.4 / 89.7
**33**	HBR_RP1_hgx1866v1_pB27_B-83	A	18S ribosomal RNA	98.7 / 85.3
**34**	HBR_RP1_hgx1866v1_pB27_B-84	A	18S ribosomal RNA	81
**35**	HBR_RP1_hgx1866v1_pB27_B-228	A	18S ribosomal RNA	91.9 / 94.2
**36**	HBR_RP1_hgx1866v1_pB27_B-188	A	18S ribosomal RNA	99.7 / 99.2
**37**	HBR_RP1_hgx1866v1_pB27_B-204	A	18S ribosomal RNA	94.3 / 100.0
**38**	HBR_RP1_hgx1866v1_pB27_B-26	A	18S ribosomal RNA	100.0 / 95.6
**39**	HBR_RP1_hgx1866v1_pB27_B-134	A	18S ribosomal RNA	98.8 / 88.4
**40**	HBR_RP1_hgx1866v1_pB27_B-145	A	18S ribosomal RNA	95.3 / 88.5
**41**	HBR_RP1_hgx1866v1_pB27_B-54	A	18S ribosomal RNA	97.7 / 92.3
**42**	HBR_RP1_hgx1866v1_pB27_B-299	A	18S ribosomal RNA	92.9 / 96.6
**43**	HBR_RP1_hgx1866v1_pB27_B-77	A	18S ribosomal RNA	95.3 / 87.3
**44**	HBR_RP1_hgx1866v1_pB27_B-173	A	18S ribosomal RNA	96.0 / 88.9
**45**	HBR_RP1_hgx1866v1_pB27_B-126	A	18S ribosomal RNA	99.7 / 99.3
**46**	HBR_RP1_hgx1866v1_pB27_B-322	A	18S ribosomal RNA	97.2 / 98.5
**47**	HBR_RP1_hgx1866v1_pB27_B-68	A	18S ribosomal RNA	100.0 / 99.7
**48**	HBR_RP1_hgx1866v1_pB27_B-96	A	18S ribosomal RNA	100.0 / 86.6
**49**	HBR_RP1_hgx1866v1_pB27_B-244	A	18S ribosomal RNA	100.0 / 99.8
**50**	HBR_RP1_hgx1866v1_pB27_B-104	A	18S ribosomal RNA	100
**51**	HBR_RP1_hgx1866v1_pB27_B-199	A	18S ribosomal RNA	94.2 / 96.4
**52**	HBR_RP1_hgx1866v1_pB27_B-320	A	18S ribosomal RNA	96.1 / 79.9
**53**	HBR_RP1_hgx1866v1_pB27_B-225	A	18S ribosomal RNA	96.1 / 99.3
**54**	HBR_RP1_hgx1866v1_pB27_B-44	A	18S ribosomal RNA	99.6 / 85.5
**55**	HBR_RP1_hgx1866v1_pB27_B-90	A	18S ribosomal RNA	97.3 / 89.5
**56**	HBR_RP1_hgx1866v1_pB27_B-163	A	18S ribosomal RNA	100.0 / 100.0
**57**	HBR_RP1_hgx1866v1_pB27_B-329	A	18S ribosomal RNA	100.0 / 98.7
**58**	HBR_RP1_hgx1866v1_pB27_B-43	A	18S ribosomal RNA	100.0 / 97.1
**59**	HBR_RP1_hgx1866v1_pB27_B-334	A	18S ribosomal RNA	93.3 / 89.1
**60**	HBR_RP1_hgx1866v1_pB27_B-100	A	18S ribosomal RNA	99.5 / 99.5
**61**	HBR_RP1_hgx1866v1_pB27_B-111	A	18S ribosomal RNA	94.7 / 96.1
**62**	HBR_RP1_hgx1866v1_pB27_B-55	A	18S ribosomal RNA	99.5 / 96.4
**63**	HBR_RP1_hgx1866v1_pB27_B-302	A	18S ribosomal RNA	96.3 / 87.4
**64**	HBR_RP1_hgx1866v1_pB27_B-324	A	18S ribosomal RNA	99.7 / 94.6
**65**	HBR_RP1_hgx1866v1_pB27_B-45	A	18S ribosomal RNA	99.7 / 98.7
**66**	HBR_RP1_hgx1866v1_pB27_B-200	A	18S ribosomal RNA	100.0 / 91.7
**67**	HBR_RP1_hgx1866v1_pB27_B-92	A	18S ribosomal RNA	100.0 / 90.8
**68**	HBR_RP1_hgx1866v1_pB27_B-69	A	18S ribosomal RNA	100.0 / 97.8
**69**	HBR_RP1_hgx1866v1_pB27_B-50	A	18S ribosomal RNA	97.9 / 93.2
**70**	HBR_RP1_hgx1866v1_pB27_B-21	A	18S ribosomal RNA	100.0 / 97.3
**71**	HBR_RP1_hgx1866v1_pB27_B-291	A	18S ribosomal RNA	89.8
**72**	HBR_RP1_hgx1866v1_pB27_B-351	A	18S ribosomal RNA	96.4 / 100.0
**73**	HBR_RP1_hgx1866v1_pB27_B-146	A	18S ribosomal RNA	97.8 / 89.4
**74**	HBR_RP1_hgx1866v1_pB27_B-260	A	18S ribosomal RNA	99.4 / 92.7
**75**	HBR_RP1_hgx1866v1_pB27_B-49	A	18S ribosomal RNA	97.0 / 90.7
**76**	HBR_RP1_hgx1866v1_pB27_B-182	A	18S ribosomal RNA	94.5 / 85.2

**77**	HBR_RP1_hgx1866v1_pB27_B-160	A	18S ribosomal RNA	90.2 / 79.8
**78**	HBR_RP1_hgx1866v1_pB27_B-256	A	18S ribosomal RNA	100.0 / 92.9
**79**	HBR_RP1_hgx1866v1_pB27_B-167	A	18S ribosomal RNA	98.9
**80**	HBR_RP1_hgx1866v1_pB27_B-60	A	18S ribosomal RNA	96.9 / 89.1
**81**	HBR_RP1_hgx1866v1_pB27_B-141	A	18S ribosomal RNA	99.8 / 98.5
**82**	HBR_RP1_hgx1866v1_pB27_B-258	A	18S ribosomal RNA	100.0 / 98.6
**83**	HBR_RP1_hgx1866v1_pB27_B-101	A	18S ribosomal RNA	100.0 / 100.0
**84**	HBR_RP1_hgx1866v1_pB27_B-374	A	18S ribosomal RNA	94.2 / 98.7
**85**	HBR_RP1_hgx1866v1_pB27_B-135	A	18S ribosomal RNA	100.0 / 99.3
**86**	HBR_RP1_hgx1866v1_pB27_B-278	A	18S ribosomal RNA	90.2 / 99.2
**87**	HBR_RP1_hgx1866v1_pB27_B-131	A	18S ribosomal RNA	95.3 / 87.8
**88**	HBR_RP1_hgx1866v1_pB27_B-280	A	18S ribosomal RNA	98.0 / 95.4
**89**	HBR_RP1_hgx1866v1_pB27_B-137	A	18S ribosomal RNA	99.7 / 96.6
**90**	HBR_RP1_hgx1866v1_pB27_B-307	A	18S ribosomal RNA	98.5 / 87.9
**91**	HBR_RP1_hgx1866v1_pB27_B-88	A	18S ribosomal RNA	92.9 / 100.0
**92**	HBR_RP1_hgx1866v1_pB27_B-339	A	18S ribosomal RNA	100.0 / 93.9
**93**	HBR_RP1_hgx1866v1_pB27_B-341	A	18S ribosomal RNA	94.8 / 85.7
**94**	HBR_RP1_hgx1866v1_pB27_B-294	A	18S ribosomal RNA	98.2 / 92.4
**95**	HBR_RP1_hgx1866v1_pB27_B-95	A	18S ribosomal RNA	92.6 / 88.4
**96**	HBR_RP1_hgx1866v1_pB27_B-233	A	18S ribosomal RNA	99.5 / 86.1
**97**	HBR_RP1_hgx1866v1_pB27_B-253	A	18S ribosomal RNA	99.7 / 87.7
**98**	HBR_RP1_hgx1866v1_pB27_B-41	A	18S ribosomal RNA	99.7 / 97.8
**99**	HBR_RP1_hgx1866v1_pB27_B-349	A	18S ribosomal RNA	100.0 / 96.8
**100**	HBR_RP1_hgx1866v1_pB27_B-370	A	18S ribosomal RNA	100

TABLE 2– USIPP PSI BLAST BRAIN EXPRESSED PROTEINS

**Sequences producing significant alignments:**
SP:DBP_HUMAN Q10586 D site-binding protein OS=Homo sapiens GN=DB...
SP:Q96HD1-2 Q96HD1 Isoform 2 of Cysteine-rich with EGF-like doma...
SP:WBP11_HUMAN Q9Y2W2 WW domain-binding protein 11 OS=Homo sapie...
SP:CREL1_HUMAN Q96HD1 Cysteine-rich with EGF-like domain protein...
TR:F8WBY3_HUMAN F8WBY3 Cysteine-rich with EGF-like domain protei...
TR:B4DMD3_HUMAN B4DMD3 cDNA FLJ58174, highly similar to WW domai...
SP:CSDC2_HUMAN Q9Y534 Cold shock domain-containing protein C2 OS...
TR:A8K2B4_HUMAN A8K2B4 cDNA FLJ77490, highly similar to Homo sap...
SP:Q9Y566-2 Q9Y566 Isoform 2 of SH3 and multiple ankyrin repeat ...
SP:Q9NP71-2 Q9NP71 Isoform 2 of Carbohydrate-responsive element-...
SP:SHAN1_HUMAN Q9Y566 SH3 and multiple ankyrin repeat domains pr...
SP:MLXPL_HUMAN Q9NP71 Carbohydrate-responsive element-binding pr...
SP:IASPP_HUMAN Q8WUF5 RelA-associated inhibitor OS=Homo sapiens ...
SP:NHSL1_HUMAN Q5SYE7 NHS-like protein 1 OS=Homo sapiens GN=NHSL...
SP:MYO9B_HUMAN Q13459 Unconventional myosin-IXb OS=Homo sapiens ...

TR:J3QK89_HUMAN J3QK89 Calcium homeostasis endoplasmic reticulum...
TR:C5HU01_HUMAN C5HU01 Carbohydrate-responsive element-binding p...
TR:B4DFC1_HUMAN B4DFC1 cDNA FLJ56060, weakly similar to FERM dom...
TR:B4DF24_HUMAN B4DF24 cDNA FLJ55324, weakly similar to FERM dom...
SP:Q6UUV9-3 Q6UUV9 Isoform 3 of CREB-regulated transcription coa...
SP:Q6UUV9-2 Q6UUV9 Isoform 2 of CREB-regulated transcription coa...
SP:ZFHX4_HUMAN Q86UP3 Zinc finger homeobox protein 4 OS=Homo sap...
SP:CRTC1_HUMAN Q6UUV9 CREB-regulated transcription coactivator 1...
TR:B4DEX7_HUMAN B4DEX7 cDNA FLJ51806, highly similar to Homo sap...
SP:NOTO_HUMAN A8MTQ0 Homeobox protein notochord OS=Homo sapiens ...
SP:YS027_HUMAN Q9H6K5 Putative uncharacterized protein FLJ22184 ...
SP:LMTK3_HUMAN Q96Q04 Serine/threonine-protein kinase LMTK3 OS=H...
SP:C102A_HUMAN Q96A19 Coiled-coil domain-containing protein 102A...
SP:Q8N3V7-2 Q8N3V7 Isoform 2 of Synaptopodin OS=Homo sapiens GN=...
SP:Q68DA7-5 Q68DA7 Isoform 5 of Formin-1 OS=Homo sapiens GN=FMN1
SP:Q5VWQ8-3 Q5VWQ8 Isoform 3 of Disabled homolog 2-interacting p...
SP:O96008-2 O96008 Isoform 2 of Mitochondrial import receptor su...
SP:FHDC1_HUMAN Q9C0D6 FH2 domain-containing protein 1 OS=Homo sa...
SP:KIF12_HUMAN Q96FN5 Kinesin-like protein KIF12 OS=Homo sapiens...
TR:Q86XL4_HUMAN Q86XL4 RLTPR protein (Fragment) OS=Homo sapiens ...
TR:Q59G17_HUMAN Q59G17 N-methyl-D-aspartate receptor subunit 2D ...
SP:NMDE4_HUMAN O15399 Glutamate [NMDA] receptor subunit epsilon-...
TR:H0YM30_HUMAN H0YM30 Formin-1 OS=Homo sapiens GN=FMN1 PE=4 SV=1
TR:B4DEJ5_HUMAN B4DEJ5 cDNA FLJ52621, moderately similar to Homo...
TR:B3KW96_HUMAN B3KW96 cDNA FLJ42619 fis, clone BRACE3015027, hi...
SP:Q8TD84-2 Q8TD84 Isoform 2 of Down syndrome cell adhesion mole...
SP:Q86UU0-4 Q86UU0 Isoform 4 of B-cell CLL/lymphoma 9-like prote...
SP:Q86UU0-3 Q86UU0 Isoform 3 of B-cell CLL/lymphoma 9-like prote...
SP:Q86UU0-2 Q86UU0 Isoform 2 of B-cell CLL/lymphoma 9-like prote...
SP:Q6ZU65-2 Q6ZU65 Isoform 2 of Ubinuclein-2 OS=Homo sapiens GN=...
SP:HS3S4_HUMAN Q9Y661 Heparan sulfate glucosamine 3-O-sulfotrans...
SP:HCN4_HUMAN Q9Y3Q4 Potassium/sodium hyperpolarization-activate...
SP:SH3R3_HUMAN Q8TEJ3 SH3 domain-containing RING finger protein ...
SP:DSCL1_HUMAN Q8TD84 Down syndrome cell adhesion molecule-like ...
SP:BCL9L_HUMAN Q86UU0 B-cell CLL/lymphoma 9-like protein OS=Homo...
SP:UBN2_HUMAN Q6ZU65 Ubinuclein-2 OS=Homo sapiens GN=UBN2 PE=1 SV=2
TR:O94794_HUMAN O94794 HRIHFB2099 protein (Fragment) OS=Homo sap...
SP:TCRG1_HUMAN O14776 Transcription elongation regulator 1 OS=Ho...
TR:A0RZB8_HUMAN A0RZB8 Diaphanous-1 OS=Homo sapiens GN=DIAPH1 PE...
SP:Q9P1A6-3 Q9P1A6 Isoform 3 of Disks large-associated protein 2...
SP:Q9BUJ2-2 Q9BUJ2 Isoform 2 of Heterogeneous nuclear ribonucleo...
SP:Q92918-2 Q92918 Isoform 2 of Mitogen-activated protein kinase...
SP:Q8WXX7-2 Q8WXX7 Isoform Short of Autism susceptibility gene 2...
SP:O94910-2 O94910 Isoform 2 of Latrophilin-1 OS=Homo sapiens GN...
SP:ZC3H4_HUMAN Q9UPT8 Zinc finger CCCH domain-containing protein...
SP:HNRL1_HUMAN Q9BUJ2 Heterogeneous nuclear ribonucleoprotein U-...
SP:AUTS2_HUMAN Q8WXX7 Autism susceptibility gene 2 protein OS=Ho...
TR:Q6ZVI0_HUMAN Q6ZVI0 cDNA FLJ42559 fis, clone BRACE3006226 OS=...

SP:LPHN1_HUMAN O94910 Latrophilin-1 OS=Homo sapiens GN=LPHN1 PE=...
SP:N4BP3_HUMAN O15049 NEDD4-binding protein 3 OS=Homo sapiens GN...
TR:J3KSA3_HUMAN J3KSA3 WAS/WASL-interacting protein family membe...
SP:WASH3_HUMAN C4AMC7 Putative WAS protein family homolog 3 OS=H...
TR:B7Z4B8_HUMAN B7Z4B8 Heterogeneous nuclear ribonucleoprotein U...
TR:B4DSU9_HUMAN B4DSU9 cDNA FLJ58421, highly similar to Cleavage...
TR:A8K6B7_HUMAN A8K6B7 cDNA FLJ37864 fis, clone BRSSN2015982 OS=...
TR:A8K222_HUMAN A8K222 cDNA FLJ78059 OS=Homo sapiens PE=2 SV=1
SP:WASH1_HUMAN A8K0Z3 WAS protein family homolog 1 OS=Homo sapie...
SP:Q9P2F8-2 Q9P2F8 Isoform 2 of Signal-induced proliferation-ass...
SP:Q8TDC3-2 Q8TDC3 Isoform 2 of Serine/threonine-protein kinase ...
SP:Q6ZRS2-3 Q6ZRS2 Isoform 3 of Helicase SRCAP OS=Homo sapiens G...
SP:P50224-2 P50224 Isoform 2 of Sulfotransferase 1A3/1A4 OS=Homo...
SP:HRH3_HUMAN Q9Y5N1 Histamine H3 receptor OS=Homo sapiens GN=HR...
SP:MLL4_HUMAN Q9UMN6 Histone-lysine N-methyltransferase MLL4 OS=...
SP:PRR12_HUMAN Q9ULL5 Proline-rich protein 12 OS=Homo sapiens GN...
SP:HCN2_HUMAN Q9UL51 Potassium/sodium hyperpolarization-activate...
SP:SI1L2_HUMAN Q9P2F8 Signal-induced proliferation-associated 1-...
SP:SHIP1_HUMAN Q92835 Phosphatidylinositol 3,4,5-trisphosphate 5...
:BRSK1_HUMAN Q8TDC3 Serine/threonine-protein kinase BRSK1 OS=H...
SP:RAPH1_HUMAN Q70E73 Ras-associated and pleckstrin homology dom...
TR:Q59FZ8_HUMAN Q59FZ8 Nebulette non-muscle isoform variant (Fra...
TR:Q13697_HUMAN Q13697 Acetylcholinesterase (Fragment) OS=Homo s...
SP:HXA10_HUMAN P31260 Homeobox protein Hox-A10 OS=Homo sapiens G...
TR:I3L0M5_HUMAN I3L0M5 Putative ATP-dependent RNA helicase DHX33...
TR:B4DIS6_HUMAN B4DIS6 cDNA FLJ56443, highly similar to Putative...
TR:B3KWF8_HUMAN B3KWF8 cDNA FLJ43009 fis, clone BRTHA2015406, hi...
SP:Q9Y4C5-2 Q9Y4C5 Isoform 2 of Carbohydrate sulfotransferase 2 ...
SP:Q9UBP0-2 Q9UBP0 Isoform 2 of Spastin OS=Homo sapiens GN=SPAST
SP:Q8IY33-5 Q8IY33 Isoform 5 of MICAL-like protein 2 OS=Homo sap...
SP:Q7Z5N4-3 Q7Z5N4 Isoform 3 of Protein sidekick-1 OS=Homo sapie...
SP:P13378-2 P13378 Isoform 2 of Homeobox protein Hox-D8 OS=Homo ...
SP:CHST2_HUMAN Q9Y4C5 Carbohydrate sulfotransferase 2 OS=Homo sa...
SP:WASF1_HUMAN Q92558 Wiskott-Aldrich syndrome protein family me...
SP:SG223_HUMAN Q86YV5 Tyrosine-protein kinase SgK223 OS=Homo sap...
SP:FA59B_HUMAN Q75VX8 Protein FAM59B OS=Homo sapiens GN=FAM59B P...
TR:Q59H23_HUMAN Q59H23 Solute carrier family 26 member 6 variant...
TR:Q59G94_HUMAN Q59G94 Zinc finger protein 207 variant (Fragment...
SP:KCC2B_HUMAN Q13554 Calcium/calmodulin-dependent protein kinas...
SP:DUS8_HUMAN Q13202 Dual specificity protein phosphatase 8 OS=H...
SP:TAF6_HUMAN P49848 Transcription initiation factor TFIID subun...
SP:CO8A1_HUMAN P27658 Collagen alpha-1(VIII) chain OS=Homo sapie...
SP:HXD8_HUMAN P13378 Homeobox protein Hox-D8 OS=Homo sapiens GN=...
SP:BAI1_HUMAN O14514 Brain-specific angiogenesis inhibitor 1 OS=...
TR:J3KNB8_HUMAN J3KNB8 Mitogen-activated protein kinase kinase k...
TR:E9PBK0_HUMAN E9PBK0 Brain-specific angiogenesis inhibitor 1 O...
TR:E7EMG0_HUMAN E7EMG0 Protocadherin-15 OS=Homo sapiens GN=PCDH1...
TR:E5KRP5_HUMAN E5KRP5 Spastin OS=Homo sapiens GN=SPAST PE=3 SV=1

TR:B4DI23_HUMAN B4DI23 cDNA FLJ60404, highly similar to Mitogen-...
SP:GRD2I_HUMAN A4D2P6 Delphilin OS=Homo sapiens GN=GRID2IP PE=2 ...
SP:Q9BYB0-2 Q9BYB0 Isoform 2 of SH3 and multiple ankyrin repeat ...
SP:Q8WWM7-8 Q8WWM7 Isoform 8 of Ataxin-2-like protein OS=Homo sa...
SP:Q8N3X1-2 Q8N3X1 Isoform 2 of Formin-binding protein 4 OS=Homo...
SP:O15054-1 O15054 Isoform 1 of Lysine-specific demethylase 6B O...
SP:A5PL33-3 A5PL33 Isoform 3 of Protein KRBA1 OS=Homo sapiens GN...
SP:A5PL33-2 A5PL33 Isoform 2 of Protein KRBA1 OS=Homo sapiens GN...
SP:K0947_HUMAN Q9Y2F5 Uncharacterized protein KIAA0947 OS=Homo s...
SP:K1522_HUMAN Q9P206 Uncharacterized protein KIAA1522 OS=Homo s...
SP:FMN2_HUMAN Q9NZ56 Formin-2 OS=Homo sapiens GN=FMN2 PE=1 SV=4
SP:ATX2L_HUMAN Q8WWM7 Ataxin-2-like protein OS=Homo sapiens GN=A...
SP:FNBP4_HUMAN Q8N3X1 Formin-binding protein 4 OS=Homo sapiens G...
SP:FOG1_HUMAN Q8IX07 Zinc finger protein ZFPM1 OS=Homo sapiens G...
TR:Q6IAP1_HUMAN Q6IAP1 HYAL3 protein OS=Homo sapiens GN=HYAL3 PE...
SP:GRIK5_HUMAN Q16478 Glutamate receptor, ionotropic kainate 5 O...
SP:KDM6B_HUMAN O15054 Lysine-specific demethylase 6B OS=Homo sap...
TR:F8TCV2_HUMAN F8TCV2 SH3 and multiple ankyrin repeat domain 3 ...
TR:B4DMQ9_HUMAN B4DMQ9 cDNA FLJ58590, highly similar to YLP moti...
SP:SOBP_HUMAN A7XYQ1 Sine oculis-binding protein homolog OS=Homo...
SP:KRBA1_HUMAN A5PL33 Protein KRBA1 OS=Homo sapiens GN=KRBA1 PE=...
SP:Q8N6N2-2 Q8N6N2 Isoform 2 of Tetratricopeptide repeat protein...
SP:Q8N350-2 Q8N350 Isoform 2 of Protein Dos OS=Homo sapiens GN=DOS
SP:Q69YN4-3 Q69YN4 Isoform 3 of Protein virilizer homolog OS=Hom...
SP:Q5T8P6-2 Q5T8P6 Isoform 2 of RNA-binding protein 26 OS=Homo s...
SP:Q5JU85-2 Q5JU85 Isoform 2 of IQ motif and SEC7 domain-contain...
SP:A4D126-2 A4D126 Isoform 2 of Isoprenoid synthase domain-conta...
SP:SOGA2_HUMAN Q9Y4B5 Protein SOGA2 OS=Homo sapiens GN=SOGA2 PE=...
SP:CECR6_HUMAN Q9BXQ6 Cat eye syndrome critical region protein 6...
SP:SALL3_HUMAN Q9BXA9 Sal-like protein 3 OS=Homo sapiens GN=SALL...
SP:MINT_HUMAN Q96T58 Msx2-interacting protein OS=Homo sapiens GN...
SP:RANB9_HUMAN Q96S59 Ran-binding protein 9 OS=Homo sapiens GN=R...
SP:MBD6_HUMAN Q96DN6 Methyl-CpG-binding domain protein 6 OS=Homo...
SP:FUBP2_HUMAN Q92945 Far upstream element-binding protein 2 OS=...
SP:T151A_HUMAN Q8N4L1 Transmembrane protein 151A OS=Homo sapiens...
SP:DND1_HUMAN Q8IYX4 Dead end protein homolog 1 OS=Homo sapiens ...
TR:Q8IV54_HUMAN Q8IV54 TSC22D4 protein OS=Homo sapiens GN=TSC22D...
SP:I2BP1_HUMAN Q8IU81 Interferon regulatory factor 2-binding pro...
TR:Q86VQ2_HUMAN Q86VQ2 WAS protein family, member 3, isoform CRA...
TR:Q53TY4_HUMAN Q53TY4 CSMD2 protein OS=Homo sapiens GN=CSMD2 PE...
SP:AFAD_HUMAN P55196 Afadin OS=Homo sapiens GN=MLLT4 PE=1 SV=3
TR:O15419_HUMAN O15419 CAGH4 alternate open reading frame OS=Hom...
SP:TNC18_HUMAN O15417 Trinucleotide repeat-containing gene 18 pr...
TR:F8WDD0_HUMAN F8WDD0 Biorientation of chromosomes in cell divi...
TR:B7Z3V9_HUMAN B7Z3V9 cDNA FLJ54490, highly similar to Protein ...
TR:B7Z3A4_HUMAN B7Z3A4 cDNA FLJ56906, highly similar to RNA-bind...
TR:B4DJ12_HUMAN B4DJ12 cDNA FLJ58355, highly similar to Tyrosine...
TR:B4DGR1_HUMAN B4DGR1 cDNA FLJ51674, highly similar to Wiskott-...

TR:B2RCD8_HUMAN B2RCD8 cDNA, FLJ96012, highly similar to Homo sa...
SP:Q9Y2G1-2 Q9Y2G1 Isoform 2 of Myelin gene regulatory factor OS...
SP:Q9NZM4-2 Q9NZM4 Isoform 2 of Glioma tumor suppressor candidat...
SP:Q9BQW3-1 Q9BQW3 Isoform 1 of Transcription factor COE4 OS=Hom...
SP:Q96PY5-3 Q96PY5 Isoform 2 of Formin-like protein 2 OS=Homo sa...
SP:Q6ZN01-3 Q6ZN01 Isoform 3 of MEF2-activating motif and SAP do...
SP:Q5T4S7-2 Q5T4S7 Isoform 2 of E3 ubiquitin-protein ligase UBR4...
SP:Q5BLP8-2 Q5BLP8 Isoform 2 of Neuropeptide-like protein C4orf4...
SP:P23246-2 P23246 Isoform Short of Splicing factor, proline- an...
SP:P17600-2 P17600 Isoform IB of Synapsin-1 OS=Homo sapiens GN=SYN1
SP:ZCHC2_HUMAN Q9C0B9 Zinc finger CCHC domain-containing protein...
SP:NEB2_HUMAN Q96SB3 Neurabin-2 OS=Homo sapiens GN=PPP1R9B PE=1 ...
SP:PP1RA_HUMAN Q96QC0 Serine/threonine-protein phosphatase 1 reg...
SP:ZNF48_HUMAN Q96MX3 Zinc finger protein 48 OS=Homo sapiens GN=...
SP:DNER_HUMAN Q8NFT8 Delta and Notch-like epidermal growth facto...
TR:Q86VG2_HUMAN Q86VG2 Splicing factor proline/glutamine-rich (P...
SP:MEGF8_HUMAN Q7Z7M0 Multiple epidermal growth factor-like doma...
SP:UBR4_HUMAN Q5T4S7 E3 ubiquitin-protein ligase UBR4 OS=Homo sa...
TR:Q59H79_HUMAN Q59H79 DNA cytosine-5 methyltransferase 3 beta i...
TR:Q2L6I0_HUMAN Q2L6I0 FB19 protein OS=Homo sapiens GN=PPP1R10 P...
SP:FAS_HUMAN P49327 Fatty acid synthase OS=Homo sapiens GN=FASN ...
SP:HD_HUMAN P42858 Huntingtin OS=Homo sapiens GN=HTT PE=1 SV=2
SP:OPRK_HUMAN P41145 Kappa-type opioid receptor OS=Homo sapiens ...
SP:SYN1_HUMAN P17600 Synapsin-1 OS=Homo sapiens GN=SYN1 PE=1 SV=3
SP:RRAS_HUMAN P10301 Ras-related protein R-Ras OS=Homo sapiens G...
SP:PER1_HUMAN O15534 Period circadian protein homolog 1 OS=Homo ...
SP:SET1A_HUMAN O15047 Histone-lysine N-methyltransferase SETD1A ...
SP:WASL_HUMAN O00401 Neural Wiskott-Aldrich syndrome protein OS=...
TR:B4DGB5_HUMAN B4DGB5 cDNA FLJ55930, highly similar to Breast c...
TR:B4DEV4_HUMAN B4DEV4 cDNA FLJ56057, highly similar to Breast c...
TR:B3KXD8_HUMAN B3KXD8 cDNA FLJ45265 fis, clone BRHIP2026346, hi...
TR:B3KX82_HUMAN B3KX82 cDNA FLJ44964 fis, clone BRAWH2016209, hi...
TR:B3KT35_HUMAN B3KT35 cDNA FLJ37565 fis, clone BRCOC2000850, hi...
TR:B2RBL9_HUMAN B2RBL9 cDNA, FLJ95582, highly similar to Homo sa...
TR:B2R7P1_HUMAN B2R7P1 cDNA, FLJ93535, Homo sapiens opioid recep...
TR:A8K180_HUMAN A8K180 cDNA FLJ76749, highly similar to Homo sap...
TR:A8K0I8_HUMAN A8K0I8 cDNA FLJ76207, highly similar to Homo sap...
SP:MEX3A_HUMAN A1L020 RNA-binding protein MEX3A OS=Homo sapiens ...
SP:Q9Y6V0-2 Q9Y6V0 Isoform 2 of Protein piccolo OS=Homo sapiens ...
:Q9UHC3-3 Q9UHC3 Isoform 3 of Acid-sensing ion channel 3 OS=Ho...
SP:Q9H0E9-4 Q9H0E9 Isoform 4 of Bromodomain-containing protein 8...
SP:Q86VM9-2 Q86VM9 Isoform 2 of Zinc finger CCCH domain-containi...
SP:Q15637-5 Q15637 Isoform 5 of Splicing factor 1 OS=Homo sapien...
SP:ROBO1_HUMAN Q9Y6N7 Roundabout homolog 1 OS=Homo sapiens GN=RO...
SP:BSN_HUMAN Q9UPA5 Protein bassoon OS=Homo sapiens GN=BSN PE=1 ...
SP:SELN_HUMAN Q9NZV5 Selenoprotein N OS=Homo sapiens GN=SEPN1 PE...
SP:MED9_HUMAN Q9NWA0 Mediator of RNA polymerase II transcription...
SP:CNTP3_HUMAN Q9BZ76 Contactin-associated protein-like 3 OS=Hom...

SP:RUSC1_HUMAN Q9BVN2 RUN and SH3 domain-containing protein 1 OS...
SP:ARX_HUMAN Q96QS3 Homeobox protein ARX OS=Homo sapiens GN=ARX ...
SP:BICD1_HUMAN Q96G01 Protein bicaudal D homolog 1 OS=Homo sapie...
SP:PIEZ1_HUMAN Q92508 Piezo-type mechanosensitive ion channel co...
SP:IBPL1_HUMAN Q8WX77 Insulin-like growth factor-binding protein...
SP:WHAMM_HUMAN Q8TF30 WASP homolog-associated protein with actin...
TR:Q8N7A3_HUMAN Q8N7A3 CDNA FLJ25866 fis, clone CBR01987 OS=Homo...
SP:MILK1_HUMAN Q8N3F8 MICAL-like protein 1 OS=Homo sapiens GN=MI...
TR:Q8N397_HUMAN Q8N397 Putative uncharacterized protein DKFZp761...
SP:MLL5_HUMAN Q8IZD2 Histone-lysine N-methyltransferase MLL5 OS=...
SP:RCOR2_HUMAN Q8IZ40 REST corepressor 2 OS=Homo sapiens GN=RCOR...
TR:Q8IYX2_HUMAN Q8IYX2 Putative uncharacterized protein (Fragmen...
TR:Q59G99_HUMAN Q59G99 Dishevelled 1 isoform a variant (Fragment...
SP:FHOD3_HUMAN Q2V2M9 FH1/FH2 domain-containing protein 3 OS=Hom...
SP:FA98C_HUMAN Q17RN3 Protein FAM98C OS=Homo sapiens GN=FAM98C P...
SP:SF01_HUMAN Q15637 Splicing factor 1 OS=Homo sapiens GN=SF1 PE...
SP:PCOC1_HUMAN Q15113 Procollagen C-endopeptidase enhancer 1 OS=...
SP:DDR1_HUMAN Q08345 Epithelial discoidin domain-containing rece...
SP:MAZ_HUMAN P56270 Myc-associated zinc finger protein OS=Homo s...
SP:ABL2_HUMAN P42684 Abelson tyrosine-protein kinase 2 OS=Homo s...
SP:HNRPL_HUMAN P14866 Heterogeneous nuclear ribonucleoprotein L ...
SP:CAC1H_HUMAN O95180 Voltage-dependent T-type calcium channel s...
SP:ALX3_HUMAN O95076 Homeobox protein aristaless-like 3 OS=Homo ...
